# Reevaluation of the role of LIP-1 as an ERK/MPK-1 dual specificity phosphatase in the *C. elegans* germline

**DOI:** 10.1073/pnas.2113649119

**Published:** 2022-01-12

**Authors:** Debabrata Das, Jacob Seemann, David Greenstein, Tim Schedl, Swathi Arur

**Affiliations:** ^a^Department of Genetics, University of Texas MD Anderson Cancer Center, Houston, TX 77030;; ^b^Department of Genetics, Cell Biology, and Development, University of Minnesota, Minneapolis, MN 55455;; ^c^Department of Genetics, Washington University School of Medicine, St. Louis, MO 63110

**Keywords:** MPK-1 ERK, LIP-1 DUSP, *C. elegans*, germline

## Abstract

The fidelity of a signaling pathway depends on its tight regulation in space and time. Extracellular signal-regulated kinase (ERK) controls wide-ranging cellular processes to promote organismal development and tissue homeostasis. ERK activation depends on a reversible dual phosphorylation on the TEY motif in its active site by ERK kinase (MEK) and dephosphorylation by DUSPs (dual specificity phosphatases). LIP-1, a DUSP6/7 homolog, was proposed to function as an ERK (MPK-1) DUSP in the *Caenorhabditis elegans* germline primarily because of its phenotype, which morphologically mimics that of a *RAS*/*let-60* gain-of-function mutant (i.e., small oocyte phenotype). Our investigations, however, reveal that loss of *lip-1* does not lead to an increase in MPK-1 activity in vivo. Instead, we show that loss of *lip-1* leads to 1) a decrease in MPK-1 phosphorylation, 2) lower MPK-1 substrate phosphorylation, 3) phenocopy of *mpk-1* reduction-of-function (rather than gain-of-function) allele, and 4) a failure to rescue *mpk-1*–dependent germline or fertility defects. Moreover, using diverse genetic mutants, we show that the small oocyte phenotype does not correlate with increased ectopic MPK-1 activity and that ectopic increase in MPK-1 phosphorylation does not necessarily result in a small oocyte phenotype. Together, these data demonstrate that LIP-1 does not function as an MPK-1 DUSP in the *C. elegans* germline. Our results caution against overinterpretation of the mechanistic underpinnings of orthologous phenotypes, since they may be a result of independent mechanisms, and provide a framework for characterizing the distinct molecular targets through which LIP-1 may mediate its several germline functions.

## Significance

The RAS–ERK pathway is critical for metazoan development. In development, ERK activity is regulated by a balance of phosphorylation of ERK by MEK (MAPK kinase) and dephosphorylation by DUSPs (dual specificity phosphatases). LIP-1, a DUSP6/7 family member, was previously suggested to regulate MPK-1/ERK activity by dephosphorylating MPK-1 in the *Caenorhabditis elegans* germline, based on LIP-1's mutant phenotype in the germline and its DUSP role in vulval development. However, our investigations demonstrate that LIP-1 does not function as an MPK-1 DUSP in the germline and likely regulates germline functions through distinct targets. Our results present a cautionary note about misinterpreting similar mutant phenotypes caused by mutations in different genes and assuming that genes function similarly in different tissues.

**Fig. 1.**
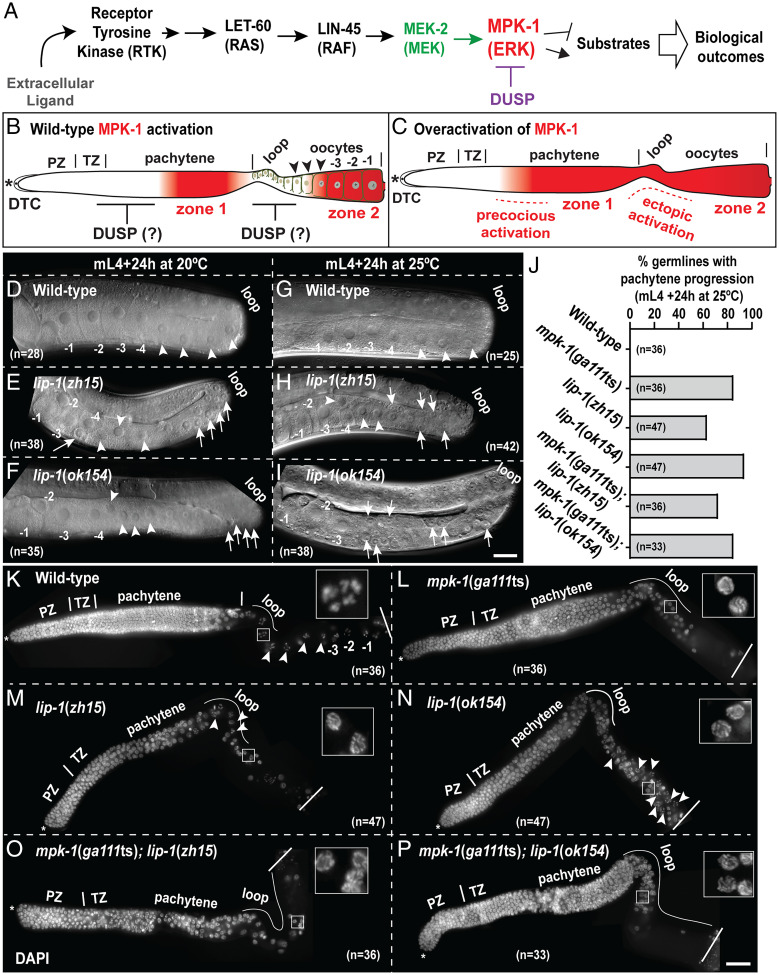
*lip-1* mutants are defective in pachytene exit and oocyte formation. (*A*) Schematic view of the conserved LET-60 (RAS)–MPK-1 (ERK) pathway showing that the regulation of ERK/MPK-1 activation depends on upstream kinase cascade and dephosphorylation depends on DUSPs. (*B*) Schematic view of a hermaphroditic *C. elegans* germline displaying the spatiotemporal nature of MPK-1 activation. The germline is oriented in a distal (*) to proximal direction from left to right. Proliferative PZ cells are in the distal region, capped by the distal tip cell (DTC). Germ cells enter meiotic prophase at the transition zone (TZ), followed by progression through different stages of meiotic prophase. The “loop region” is the anatomic bend in the U-shaped gonad. The −1 marks the oldest oocyte at the proximal end. Active MPK-1 is visualized by a specific dpMPK-1 antibody in two distinct regions of the germline: midpachytene, termed as zone 1, and proximal few oocytes, termed as zone 2. The intensity of the color (red) correlates with strength of MPK-1 di-phosphorylation. (*C*) Predicted activation of MPK-1 in the absence of DUSP: either distal to zone 1, called “precocious” activation, or in the late-pachytene/early-diplotene region (anatomically in the loop region), called “ectopic” activation. (*D*–*I*) Differential interference contrast microscopy images of germlines from indicated genotypes, age, and temperature to visualize germline morphology. The loop region is on the right in the photographs and oocytes on the ventral side. Oocytes are numbered from proximal to distal polarity (toward loop). The most proximal oocyte is labeled as −1. Arrowheads indicate oocytes, and arrows indicate pachytene-stage germ cells. (*J*) Quantification of germlines of the indicated genotypes, with pachytene-progression–defective phenotypes expressed as a percentage. (*K*–*P*) Dissected DAPI-stained germlines of the indicated genotypes (mid-L4 + 24 h at 25 °C) displaying germline morphology. Insets are magnified views of germ cell(s) at the proximal gonad (after loop region). The dissected germlines are oriented with the distal on the left (*) to proximal on the right of the photograph, according to the meiotic progression. Arrowheads indicate oocytes, and arrows indicate pachytene germ cells. The total number of germlines (*n*) analyzed per genotype is indicated in each panel (scale bars, 25 μm).

Extracellular signal-regulated kinases (ERKs) are a group of serine/threonine protein kinases and classical members of mitogen activated protein kinases (MAPKs). The ERK MAPKs are terminal enzymes of a highly conserved three-tiered kinase signaling cascade, the RAS–ERK pathway (1, 2). Extracellular stimuli, including growth factors and insulin signaling induce the sequential activation of RAS–ERK pathway that orchestrates a wide range of cellular processes such as gene expression, proliferation, differentiation, and apoptosis to regulate tissue and organismal homeostasis (Fig. 1*A*) (1–3). Because the ERK MAPK signaling pathway regulates a myriad of developmental processes for controlled and ordered execution of the pathway, ERK activity is tightly monitored in space and time (4). MEK (also known as MAPK/ERK kinase) phosphorylates ERK at threonine and tyrosine residues (TEY motif), thus activating its function (1). Active ERK is then inactivated by dual specificity MAPK phosphatases (MKPs or DUSPs) that remove the phosphate residues. Together, MEK and DUSPs shape the magnitude, duration, and spatiotemporal profile of ERK activity (1, 4–6).

The *Caenorhabditis elegans* oogenic germline, like most complex biological systems, displays a controlled spatiotemporal pattern of ERK (MPK-1 in *C. elegans*) activity (7–11). Active MPK-1, as assayed using an antibody that detects dual phosphorylated MPK-1 at threonine and tyrosine of the TEY motif (7, 12), is visualized in midpachytene (termed as zone 1 of activation). However, MPK-1 is dephosphorylated, and thus, its activity is very low in the late-pachytene and early-diplotene region of the germline, which corresponds to the anatomic “loop” of the *C. elegans* U-shaped gonad (Fig. 1*B*). MPK-1 phosphorylation is again visible in the proximal diakinesis oocytes (termed as zone 2) in a hermaphroditic germline (7–9). Zone 2 activation is mediated by a secreted sperm signal (major sperm protein, or MSP), which antagonizes the VAB-1 Ephrin receptor (13). Thus, zone 2 activation is absent in *C. elegans* females, which do not produce sperm (7). In a wild-type oogenic hermaphroditic germline, active MPK-1 has not been visualized in the distal germline, from the progenitor zone (PZ) to midpachytene, and is very low in the loop region of the germline. Because total MPK-1 protein is expressed throughout the germline (8), the striking spatiotemporal activation pattern of MPK-1 observed using the dual-phosphospecific antibody suggests localized activation and inactivation of MPK-1 through MEK and DUSPs.

In the oogenic hermaphroditic germline, the phenotypic consequences of MPK-1 activation are complex. In genetic mutants of the *mpk-1* pathway, changes to the MPK-1 activation pattern along the spatiotemporal axis, as well as alterations to signal strength, produce distinct phenotypes. For example, a complete loss of MPK-1 activity in a null allele causes the oogenic germ cells to arrest in early- to midpachytene (8, 14). In the absence of MPK-1 activity, the germ cells fail to launch the apoptotic program because they do not progress into midpachytene, the stage in which meiotic checkpoint activation culls errors (9, 15). Reduction of MPK-1 signal strength using temperature-sensitive (ts) alleles, however, produced different phenotypes depending on the time at which MPK-1 activation was reduced during oogenic development. These *mpk-1*(ts) germlines exhibit increased apoptosis (due to higher levels of meiotic asynapsis defects; Ref. 11), a pachytene-progression defect in which pachytene-stage cells linger and are observed in the loop region, and fewer oocytes with an increased size relative to wild-type animals (8). Conversely, in *RAS*/*let-60* gain-of-function mutants, the spatiotemporal pattern of MPK-1 activation is different from the wild-type in two regions: 1) in midpachytene, the germline displays “precocious” activation of MPK-1, and 2) the loop region exhibits “ectopic” MPK-1 activation (Fig. 1*C*). These animals, unlike the wild-type, display multiple small oocytes (8). Because of the striking increase in oocyte number in the *RAS*/*let-60* gain-of-function mutants, an increase in oocyte number has been considered as a readout for MPK-1 activation. Mutants displaying multiple small oocytes are thus interpreted to be a consequence of increased MPK-1 activity.

The *C. elegans* genome has 29 predicted DUSPs, of which LIP-1 (lateral signal induced phosphatase-1) bears homology with mammalian DUSP6/7 (16, 17). Genetic evidence suggested that loss of *lip-1* negatively regulates MPK-1 during somatic vulval development (17). In vitro, in mammalian Cos-1 cultured cells, Myc-tagged LIP-1 protein was shown to dephosphorylate mammalian ERK1/2 (16). Coupled with the homology to mammalian DUSPs, the authors concluded that LIP-1 functions as an MPK-1 DUSP in vivo. In the *C. elegans* germline, immunofluorescence staining using an anti-LIP-1 antibody showed that the total protein is expressed from the proximal one-third of the PZ region and throughout the pachytene as membrane-associated bright puncta (18). LIP-1 was proposed to function as an MPK-1 DUSP, in the germline, from two lines of evidence (18), which we reevaluated based on the reasoning outlined below. First, in the prior report, the authors showed that in a feminized germline, which does not produce any sperm signal, loss of *lip-1* led to an increase in phosphorylated MPK-1 in zone 2 (Fig. 1*B*). However, in the absence of the sperm signal, MPK-1 cannot be phosphorylated in zone 2 to begin with (7, 13) (Fig. 1*A*). In this context, inactivation or absence of a DUSP (LIP-1, in this case) should not lead to an increase in the level of phosphorylated MPK-1 since it was never phosphorylated. Second, the authors observed that loss of *lip-1* led to ectopic (loop region) MPK-1 activation in hermaphrodites coupled with an increase in oocyte numbers. The authors interpreted this phenotype to be similar to that of *RAS*/*let-60* gain-of-function mutant germlines (18). However, recent work has revealed that the increased oocyte production in *RAS*/*let-60* gain-of-function animals is due to the “precocious” activation of MPK-1 in the early-pachytene, rather than the “ectopic” MPK-1 activation in the loop region (Fig. 1*C*) (11). Together, these two lines of reasoning led us to reinvestigate the role of LIP-1 as an MPK-1 DUSP in the *C. elegans* germline and to determine where in the germline spatiotemporal axis LIP-1 might function to regulate oocyte formation, using cytology, genetics, and phenotypic analyses.

Contrary to what was previously published (18), our results show that 1) precocious or ectopic MPK-1 activity is not detected in the absence of *lip-1*—in fact, we found that loss of *lip-1* led to lower MPK-1 activation; 2) loss of *lip-1* fails to rescue the pachytene-progression and fertility defects observed upon reducing *mpk-1* function; and 3) germlines with loss of *lip-1* displayed an *mpk-1* loss-of-function–like oocyte phenotype, rather than a gain-of-function–like oocyte phenotype, and 4) led to lower MPK-1 substrate phosphorylation. Moreover, we show that mutants in other genes, such as *ooc-5* (human ortholog of torsinA AAA+ ATPase), also exhibit multiple small oocytes (19, 20) but do not present with ectopic MPK-1 activity, suggesting that increased oocyte number is not invariably equivalent to, or due to, increased MPK-1 phosphorylation. In support of this, we observed that loss of *rskn-1* (human ortholog of *RPS6KA*, ribosomal protein S6 kinase A), which results in increased ectopic activation of MPK-1 in the loop region of the germline, does not exhibit increased oocyte numbers. This finding demonstrates that ectopic MPK-1 activation does not necessarily cause oocyte numbers to increase. Finally, in wild-type *C. elegans* diplotene oocytes, the synaptonemal complex (SC) central proteins are removed from the long arm of the chromosome axis to allow for accurate chromosome segregation (21). However, RAS/*let-60*(*ga89*ts) gain-of-function mutants have been shown to retain the SC central proteins on the long arm (10). Nadarajan et al. (10) reported that loss of *lip-1* also leads to retention of the SC central protein to the long chromosomal arm and proposed that this was because of an increase in MPK-1 activation. We found that while the SC central element proteins are retained on the long arm of the chromosome in diplotene oocytes in both RAS/*let-60*(*ga89ts*) gain-of-function and *lip-1* mutant oocytes, they are not retained in the *rskn-1* mutant germlines, which display increased MPK-1 activation in oocytes. Thus, the retention of the SC central proteins in *lip-1* mutant germlines likely occurs through MPK-1–independent mechanisms, suggesting that multiple regulatory processes, both independent of and dependent on ectopic MPK-1 phosphorylation, control SC disassembly. Together, these data demonstrate that LIP-1 does not function as an MPK-1 DUSP in the context of the *C. elegans* germline and may have multiple other targets through which it mediates its several germline functions.

## Results and Discussion

### LIP-1 Promotes Pachytene Progression and Regulates Oocyte Growth in a Temperature-Sensitive Manner.

To reinvestigate the role of LIP-1 as an MPK-1 DUSP in the *C. elegans* germline, we used two different *lip-1* alleles: 1) the previously characterized deletion allele *lip-1*(*zh15*) (17) and 2) *ok154*, which bears a deletion of 1504 base pairs (bp) that starts from −156 bp and ends at +1348 bp, deleting the start codon; we call this the “*null*” allele (*SI Appendix*, Fig. S1). If LIP-1 functions as an MPK-1 DUSP, then loss of *lip-1* should result in increased MPK-1 activation, which should in turn rescue (or ameliorate) the phenotypes caused by an MPK-1 reduction-of-function mutation such as *mpk-1*(*ga111*ts). *ga111* is a temperature-sensitive mutation in the MEK-binding site on MPK-1 (22), which presents with a reduction in MPK-1 phosphorylation (8, 9, 11). An absence (or reduction) of dephosphorylation in *lip-1* mutants should enable the perdurance of MPK-1 phosphorylation and thus lead to phenotypic rescue of the *mpk-1* reduction-of-function allele. Instead, we observed that 1) loss of *lip-1* in the *mpk-1*(*ga111*ts) background does not rescue the *mpk-1* mutant phenotypes and, more surprisingly, 2) the double mutants between *mpk-1*(*ga111*ts) and *lip-1*, both at 20 and 25 °C, appear indistinguishable from *mpk-1* single mutants at 20 and 25 °C. Together these data suggest that *lip-1* does not function as a negative regulator of *mpk-1* and that, in fact, *mpk-1* may be epistatic to *lip-1* rather than vice versa. Below (in the next three paragraphs), we describe the data that supports these conclusions.

We examined the germline phenotypes of the *lip-1* single mutants and the *lip-1* alleles with *mpk-1*(*ga111*ts) as double mutants at 20 and 25 °C. As published previously (18), *lip-1* mutants display disorganized oocytes at 20 °C, unlike wild-type animals, which display a linear row of oocytes (Fig. 1 *D*–*F*). However, we discovered that *lip-1* mutants show temperature sensitivity, such that the disorganized oocyte phenotype is more severe at 25 °C in both *lip-1* alleles (Fig. 1 *G*–*I*). Because this “temperature sensitivity” is observed in *lip-1* null alleles, it must reflect a temperature-dependent process that is revealed in the absence of *lip-1*, rather than a temperature-sensitive protein product. In addition, in live whole-mounts, as well as in dissected gonadal preparations of both the *lip-1* alleles at 20 and 25 °C, we observed the presence of pachytene-like germ cells in the proximal gonad, with a more severe penetrance at 25 °C (Fig. 1 *E*–*N*). In wild-type germlines, pachytene germ cells transit to diplotene stage just distal to the loop region (Fig. 1*K*). However, in the *lip-1* mutant alleles, pachytene-stage germ cells are observed proximal to the loop region (∼62% in *zh15* and ∼93% in *ok154* allele at 25 °C; Fig. 1 *J*, *M*, and *N*, white box), a phenotype defined as a pachytene-progression defect (9) (Fig. 1*J*). This phenotype is similar to that observed in *mpk-1*(*ga111*ts) germlines at 25 °C (∼83%, *n* = 36) (Fig. 1* J* and* L*). A pachytene-progression defect was unexpected since loss of an MPK-1 DUSP should not mimic loss of MPK-1.

At 25 °C, *mpk-1*(*ga111*ts) single mutants are ∼90% sterile, and fertile animals exhibit very high embryonic lethality (*SI Appendix*, Fig. S2 *A* and *B*). We observed that the double mutants between *lip-1* and *mpk-1*(*ga111*ts) [*mpk-1*(*ga111*ts)*; lip-1*(*zh15*) and *mpk-1*(*ga111*ts)*; lip-1*(*ok154*)] failed to suppress the *mpk-1*(*ga111*ts) pachytene-progression defect and sterility at 25 °C (Fig. 1 *J*, *O*, and *P*, white box; *SI Appendix*, Fig. S2 *A* and *B*), unlike what was reported previously (18). In fact, while the penetrance of the pachytene-progression defect was similar between *lip-1* single mutants and *mpk-1*(*ga111*ts)*; lip-1* double mutants (Fig. 1*J*), morphologically, the germlines of the double mutants appeared indistinguishable from the *mpk-1*(*ga111*ts) single mutants (Fig. 1; compare Fig. 1*L* to Fig. 1*P*).

We observed that not only did loss of *lip-1* fail to suppress *mpk-1*(*ga111*ts) sterility at 25 °C (*SI Appendix*, Fig. S2 *A* and *B*), but loss of *lip-1* enhanced the *mpk-1*(*ga111*ts) subfertility phenotype at 20 °C (*SI Appendix*, Fig. S2 *C* and *D*). Although the *mpk-1*(*ga111*ts) single mutant produces ∼130 to 270 progeny at 20 °C, when combined with the *lip-1* mutant alleles, the *mpk-1*(*ga111*ts) double mutants generate fewer progeny, ranging from 10 to 250 per hermaphrodite at 20 °C (*SI Appendix*, Fig. S2*C*). This subfertility was coupled with an increase in embryonic lethality in the double mutants compared to the embryonic lethality in *mpk-1*(*ga111*ts) single mutants (*SI Appendix*, Fig. S2*D*). These data demonstrate that both the *lip-1* alleles enhanced *mpk-1*(*ga111*ts) infertility at 20 °C, leading to a reduction in progeny numbers. However, the oocytes from the *mpk-1*(*ga111*ts)*; lip-1* double mutants appeared to be arranged in a linear row similar to the *mpk-1*(*ga111*ts) single mutant alleles at 20 °C (*SI Appendix*, Fig. S3). These data indicate that *mpk-1*(*ga111*ts) suppresses the *lip-1* disorganized oocyte phenotype at 20 °C. Taken together, these observations suggest a complex genetic interaction between *lip-1* and *mpk-1*, which is inconsistent with the notion that the LIP-1 simply inhibits MPK-1 activity.

One reason for the discrepancy of the data reported here and the prior report (18) could be because the prior study analyzed the wild-type and *lip-1* mutants at 20 °C but analyzed the double mutants between *lip-1* and *mpk-1* only at 25 °C. However, given that *lip-1* mutations exhibit temperature-dependent phenotypes, comparisons should be conducted at the same temperature to provide a more comprehensive picture. Together, we conclude that loss of *lip-1* does not rescue loss of *mpk-1*–mediated fertility defects.

### LIP-1 Promotes, Rather Than Inhibits, MPK-1 Activation in the Germline.

**Fig. 2.**
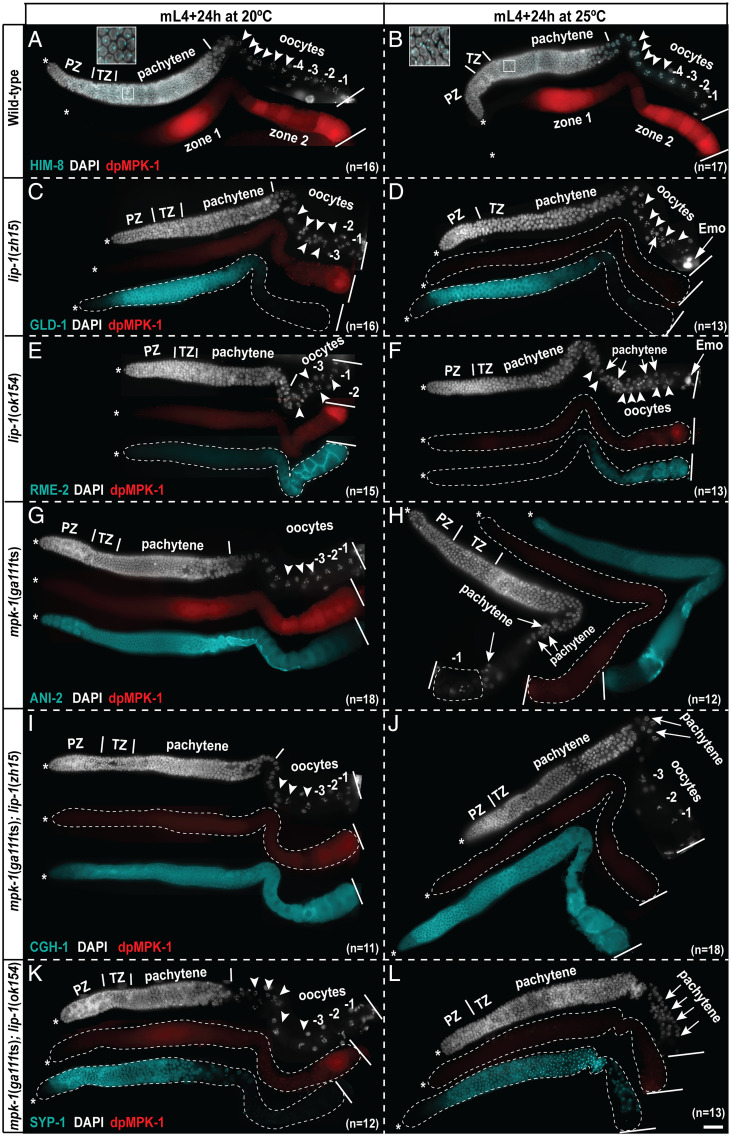
*lip-1* promotes MPK-1 activity in the germline. (*A*–*L*) Representative images of dissected germlines probed with anti–dpMPK-1 (red), genotype marking antibodies (cyan), and DAPI (DNA, white) for the indicated genotypes, ages, and temperatures. The HIM-8 panel is overlapped with DAPI, and an enlarged view of the early-pachytene region is shown in the inset. The dissected germlines are displayed in a distal (*Left*, *) to proximal (*Right*) orientation. Oocytes are numbered from proximal to distal polarity (toward loop). The most proximal oocyte is labeled as −1. Arrowheads indicate oocytes, and arrows indicate pachytene germ cells. The total number of germlines (*n*) analyzed per genotype is indicated in each panel, all showing similar dpMPK-1 staining patterns (scale bar, 25 μm).

**Fig. 3.**
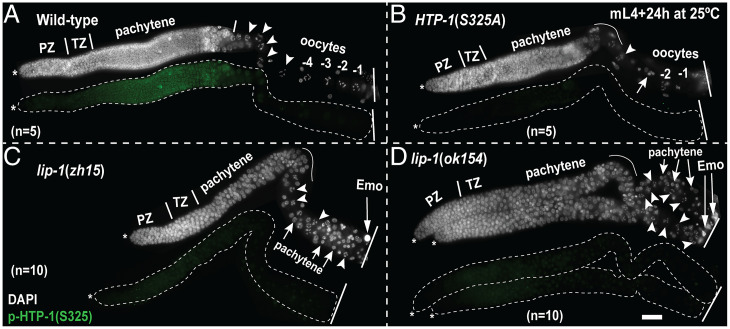
*lip-1* mutants exhibit reduced phosphorylation of an MPK-1 substrate in the germline. (*A*–*D*) Representative images of dissected germlines probed with anti-p-HTP-1(S325) (green) and DAPI (DNA, white) of indicated genotypes. The dissected germlines are displayed in a distal (*Left*, *) to proximal (*Right*) orientation. Oocytes are numbered from proximal to distal polarity (toward loop). The most proximal oocyte is labeled as −1. Arrowheads indicate oocytes, and arrows indicate pachytene germ cells. The total number of germlines (*n*) analyzed per genotype is indicated in each panel (scale bar, 25 μm).

A phosphatase functions to remove the phosphate group on a protein. Thus, loss of a phosphatase should lead to retention of the phosphorylation or a net increase in phosphorylation in the presence of an upstream activating kinase. MPK-1 phosphorylation is detected using a monoclonal antibody that specifically recognizes the di-phosphorylated (on the TEY motif) form of MPK-1 (dpMPK-1) (7–9, 11, 12). LIP-1 has been described to function as a DUSP in vivo and should remove both the phosphorylation sites on MPK-1. Thus, loss of *lip-1* was thought to lead to an increase in dpMPK-1 levels in the germline. However, we observed that loss of *lip-1* did not lead to an increase in dpMPK-1 levels and instead, and surprisingly, led to a decrease in dpMPK-1 levels in the germline (Fig. 2). This decrease in dpMPK-1 signal levels was mirrored by a decrease in the phosphorylation of an MPK-1 substrate (Fig. 3). We also observed that in the absence or presence of the sperm signal, in a feminized germline, loss of *lip-1* does not lead to an increase in dpMPK-1 in oocytes (*SI Appendix*, Figs. S4 and S5).

To test whether loss of *lip-1* alters the spatiotemporal pattern of MPK-1 activation in the *C. elegans* germline, we performed dpMPK-1 immunostaining on dissected germlines. To reduce the variability in staining and imaging conditions, we first labeled each of the genotypes with a different antibody and then combined the labeled germlines in one tube, followed by dpMPK-1 labeling in the same tube. The germlines were then mounted and imaged on the same slide with identical acquisition times (*Materials and Methods*). In so doing, we did not observe any precocious or ectopic activation of MPK-1 in *lip-1* mutants in the progenitor region, early-pachytene region, or in the loop region at either of the temperatures tested (Fig. 2 *A*–*F*). Rather, we found that both the *lip-1* mutant alleles displayed reduced dpMPK-1 signal in zone 1 and zone 2 at 20 °C, relative to wild-type germlines (Fig. 2 *A*, *C*, and *E*). The reduction in dpMPK-1 levels appeared more severe at 25 °C, in that dpMPK-1 was not visible at all in the *lip-1* mutant germlines (Fig. 2 *B*, *D*, and *F*). dpMPK-1 levels were low in *mpk-1*(*ga111*ts) at 20 °C, with no detectable staining at 25 °C (Fig. 2 *G* and *H*) relative to the wild-type, consistent with previous observations (8, 11).

The *mpk-1*(*ga111*ts)*; lip-1* double mutants displayed even lower dpMPK-1 signal compared to *mpk-1*(*ga111*ts) single mutants at 20 °C, while at 25 °C, the dpMPK-1 signal was below detectable levels (Fig. 2 *I*–*L*). These observations are consistent with the phenotypic analyses presented previously that loss of *lip-1* enhances *mpk-1*(*ga111*ts) mutant phenotypes rather than suppresses them. Together, these data demonstrate that LIP-1 does not function as an MPK-1 DUSP in the *C. elegans* germline; rather, it appears that LIP-1 may promote MPK-1 signaling, likely indirectly.

To further test our observation that loss of *lip-1* results in a decrease rather than an increase in MPK-1 activation, we assessed the phosphorylation of an MPK-1 substrate. We analyzed the phosphorylation status of one of the germ cell–specific MPK-1 substrates, HTP-1, an axial element protein of the SC (11). Active MPK-1 phosphorylates HTP-1 on the S325 residue. Phosphospecific HTP-1(S325) antibody detects the phosphoprotein in germ cells (Fig. 3*A*) (11). The specificity of the antibody was confirmed using the *htp-1*(*viz62*) [*HTP-1*(*S325A*)] unphosphorylatable mutant (Fig. 3*B*) (11). In the wild-type germline, phosphorylated HTP-1(S325) accumulates from the transition zone, pachytene, and into diplotene-stage oocytes in the nucleoplasm and along the chromosomes (Fig. 3*A*). However, we observed that in both the *lip-1* mutants, accumulation of p-HTP-1(S325) was lower compared to the wild-type (Fig. 2 *A*–*D*). These data indicate that loss of LIP-1 not only results in a lower dpMPK-1 signal in the germline but that the activity of MPK-1, as measured through the phosphorylation status of a substrate, is also decreased in *lip-1* mutant germlines.

Next, we tested whether loss of *lip-1* results in increased MPK-1 activation in feminized germlines as reported (18). Because feminized germlines do not produce any sperm, the dpMPK-1 signal is not detected in oocytes because of a failure to engage the EphR–RAS–ERK pathway (Fig. 1*A*; *SI Appendix*, Fig. S4) (7, 13). Consistent with lack of MPK-1 activation in oocytes, the oocytes remain arrested and consequently stack in the germline. During this time, the stem cell proliferation slows down, and the overall process of oogenesis decelerates (23, 24), also resulting in a lack of dpMPK-1 in zone 1 (7, 8). Concordant with these data, we observed that female germlines, analyzed 24 h after the mid-L4 larval stage, do not exhibit dpMPK-1 in either zone 1 or 2 (*SI Appendix*, Fig. S4 *A* and *B*). To determine whether loss of *lip-1* leads to an increase in MPK-1 phosphorylation, we assessed active MPK-1 in feminized *lip-1* germlines in the absence and presence of sperm signal. We observed that in unmated *lip-1* females, dpMPK-1 staining was not detectable in either zone 1 or zone 2 (*SI Appendix*, Fig. S4*C*), in contrast to the previous report (18). To test whether the feminized *lip-1* mutants could activate dpMPK-1 upon addition of the sperm signal, we mated the control and *lip-1* feminized animals with wild-type males (*SI Appendix*, *Methods*). Mated feminized *lip-1* mutant germlines displayed a very weak dpMPK-1 signal restricted to the −1 oocyte (*SI Appendix*, Fig. S5*B*), unlike mated control feminized germlines, which displayed robust dpMPK-1 signal in the germline (*SI Appendix*, Fig. S5*A*). Together, these data suggest that loss of *lip-1* does not lead to an increase of MPK-1 activation in the feminized germline in either absence or presence of sperm signal.

In these feminized germlines, however, the oocytes remain arrested in the diakinesis stage before fertilization. A failure of an oocyte to maintain the meiotic prophase arrest in *C. elegans* leads to onset of endoreduplication cycles resulting in an endomitotic oocyte (Emo) phenotype (8, 25, 26). These highly polyploid oocytes with enlarged nuclei are visible as “blobs” of DNA upon labeling with DAPI (compare *SI Appendix*, Fig. S6 *A* and *B*). As we report above, *lip-1* mutations confer temperature-dependent mutant phenotypes. To observe only the impact of loss of *lip-1* in a feminized background, we chose to use a feminizing null allele [*fog-2*(*q71*)], which is not temperature dependent (27), such that we could compare the feminized *lip-1* mutant at 20 and 25 °C (*SI Appendix*, Fig. S6 *C* and *D*). We observed that *lip-1* females display ∼40 and ∼80% Emo phenotype at 20 and 25 °C, respectively (*SI Appendix*, Fig. S6 *C* and *D*), relative to *fog-2*(*q71*) single mutants, which display ∼4.5% Emo phenotype at both 20 and 25 °C (*SI Appendix*, Fig. S6 *C* and *D*). The high level of Emo phenotype at 20 °C in *lip-1* females was not previously reported (18) because the authors had conducted the experiment using two distinct temperature-sensitive mutants [*fog-1*(*q253*ts) or *fem-2*(*b245*ts)], which display feminization only at 25 °C. We interpret the high level of Emo phenotype even at 20 °C in *lip-1* females to mean that the *lip-1* mutants are defective in maintaining meiotic prophase arrest. However, given that we did not observe reactivation of dpMPK-1 in the proximal oocytes of *lip-1* mutants, the presence of the Emo phenotype cannot be attributed to reactivation of MPK-1. Moreover, the disorganized, small oocytes observed in the feminized *lip-1* mutant germline (*SI Appendix*, Figs. S4*C* and S6*B*) are distinct from those observed in feminized *let-60*(*ga89*ts) germlines, which are more linearly arrayed in the germline and more uniform in size (8, 28). Thus, LIP-1 likely acts through MPK-1–independent mechanisms to regulate oocyte meiotic prophase arrest in the female germline. Together with the phenotypic analysis, dpMPK-1 staining pattern, and MPK-1 substrate phosphorylation analysis, these data indicate that LIP-1 does not function as an MPK-1 DUSP; rather, it may indirectly activate the pathway in the *C. elegans* germline.

### Small Oocyte Phenotypes Do Not Necessarily Indicate an Increase in MPK-1 Activation.

A well-characterized phenotype for overactivation of MPK-1 pathway is the formation of multiple disorganized, small oocytes in the *C. elegans* germline (8, 9, 11, 28). In the literature, a small oocyte phenotype is almost always thought to be a result of ectopic and hyperactivation of MPK-1 in the loop region. Mutants displaying multiple small oocytes are thus interpreted to lead to an increase in MPK-1 activity. However, *let-60*(*ga89*ts) mutants display an increase in dpMPK-1 levels, relative to wild-type, even at 20 °C (8, 11). Yet, at 20 °C, these germlines display a linear and “normal” oocyte pattern, suggesting that an ectopic activation of MPK-1 does not necessarily indicate that oocyte numbers will be higher. In addition, we recently discovered that the “precocious” activation of MPK-1 in the early-pachytene region, rather than an “ectopic” activation in the loop region, promotes faster meiotic progression in the *RAS*/*let-60* gain-of-function mutant, which in turn leads to the earlier pachytene exit of germ cells and the formation of multiple small oocytes (11). These observations together led us to ask whether there were mutations in genes that lead to an increase in oocyte number but that do not affect MPK-1 activation and vice versa—mutations that increase dpMPK-1 levels but do not affect oocyte numbers.

**Fig. 4.**
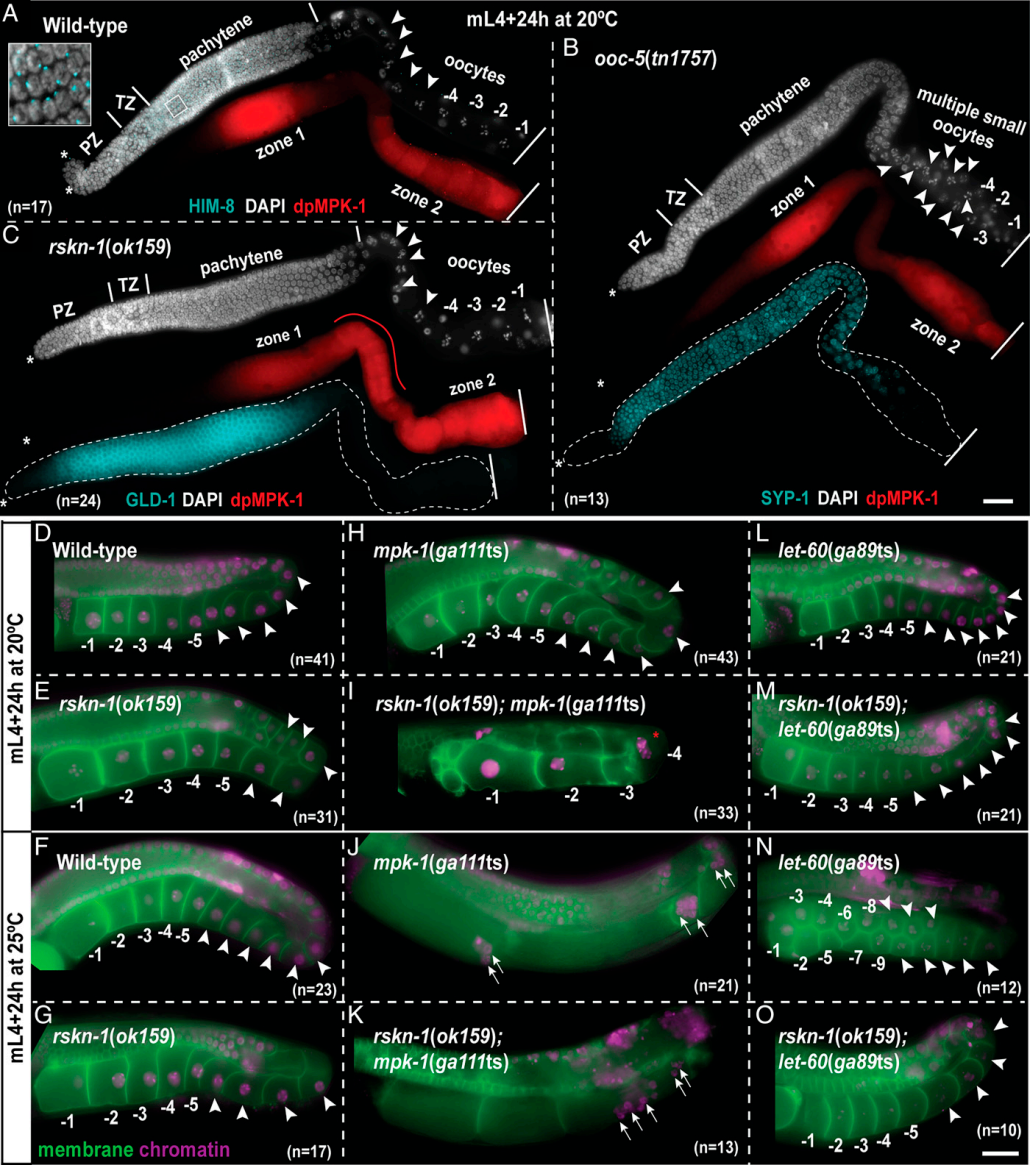
Ectopic MPK-1 activation in the loop region does not necessarily lead to an increase in oocyte production. (*A*–*C*) Representative images of dissected germlines probed with anti–dpMPK-1 (red), genotype marking antibodies (cyan), and DAPI (DNA, white) for indicated genotypes at 20 °C. The dissected germlines are displayed in a distal (*Left*, *) to proximal (*Right*) orientation. A red line highlights the ectopic MPK-1 activation in the *rskn-1*(*ok159*) germline. The HIM-8 panel is overlapped with DAPI, and an enlarged view of the early-pachytene region is shown in the *Inset*. (*D*–*O*) Representative live-fluorescent images for the indicated genotypes and temperatures, displaying germline morphology marked by pH domain membrane green fluorescent protein (GFP; green) and histone H2B::mCherry (magenta). Oocytes are numbered from proximal to distal polarity (toward loop). The −1 marks the oldest oocyte at the proximal end. A red asterisk marks a binucleate oocyte. Arrowheads indicate oocytes, and arrows indicate pachytene germ cell. The total number of germlines (*n*) analyzed per genotype is indicated in each panel; all showed similar dpMPK-1 staining pattern (scale bars, 25 μm).

To determine whether precocious or ectopic activation of MPK-1 underlies small oocyte phenotypes in distinct pathway mutants, we assayed the MPK-1 activation pattern in *ooc-5*(*null*) animals. *ooc-5*(*null*) animals exhibit an increased number of small oocytes, a phenotype that morphologically appears identical to a *RAS*/*let-60* gain-of-function germlines (8, 19, 20). We observed the dpMPK-1 signal is neither precocious in the early-pachytene region nor ectopic through the loop region (diplotene oocytes) in *ooc-5*(*null*) germlines. In fact, the dpMPK-1 signal was similar to the wild-type in these germlines (Fig. 4 *A* and *B*), suggesting that the small oocyte phenotype is not necessarily correlated with precocious or ectopic activation of MPK-1. In addition, multiple lines of evidence in the field suggest that oocyte number and size are regulated by distinct parallel pathways, involving different cellular processes (8, 29, 30). For example, loss of GLP-1 Notch signaling causes a reduction in oocyte number coupled with formation of a large oocyte (29). This large oocyte phenotype is remarkably similar to the *mpk-1*(*ga111*ts) mutants (8, 29). However, the cause of the phenotype is distinct in these two mutants. In the *mpk-1*(*ga111*ts) mutants, a slower rate of pachytene progression causes fewer germ cells to advance on to the diplotene/diakinesis stage to form oocytes (11), whereas loss of Notch signaling is marked by lower progenitor cell number, elevated rate of cytoplasmic flow, and delayed rate of oocyte cellularization causing the phenotype (29). Further, a large phenotypic analysis of germline genes in worms by Green et al., 2009 (30) identified distinct genes and pathways that can lead to similar oocyte phenotypes. For example, RNA interference (RNAi) of genes involved in RNA metabolism and vesicle trafficking led to increased number of small oocytes (30). Thus, multiple divergent mechanisms can regulate oocyte number and growth, independent of MPK-1 signaling.

### Ectopic MPK-1 Activation in the Loop Region Does Not Necessarily Lead to an Increased Production of Oocytes.

To next determine whether an increase in ectopic dpMPK-1 signal always results in increased oocyte numbers, we focused on mutants that caused an ectopic increase in dpMPK-1. We had previously documented MPK-1 substrates that regulate MPK-1 activation in the germline through feedback mechanisms (9). We discovered RSKN-1, a homolog of p90 RSK as a negative regulator of MPK-1 activation; loss of *rskn-1* through RNAi resulted in increased and ectopic MPK-1 activation in the loop/diplotene region (9). Here, we assessed MPK-1 activation using the *rskn-1*(*ok159*) deletion allele, which deletes 1,076 bp and causes a frameshift, thereby introducing a premature stop codon that eliminates the MPK-1 docking site and other functional domains in RSKN-1 (*SI Appendix*, Fig. S7 *A*–*C*). We observed that, as with the RNAi analysis (9), *rskn-1*(*ok159*) germlines displayed ectopic MPK-1 activation in the loop region, but not precocious activation in early pachytene (Fig. 4*C*), suggesting that RSKN-1 negatively regulates MPK-1 activity in the loop region. However, we did not observe any defects in oocytes at any temperature tested in this mutant (20 and 25 °C); thus, *ok159* is not a temperature-dependent allele (Fig. 4 *D*–*G*; *SI Appendix*, Fig. S7 *D* and *E*). These data further support the model that ectopic MPK-1 activation in the loop/diplotene region does not necessarily lead to an increase in oocyte number.

Moreover, in the functional genomic screen from which *rskn-1* was isolated, we had identified *rskn-1* as an enhancer of a *mpk-1* loss-of-function mutant (9), which is unexpected for a gene whose loss leads to an increase in dpMPK-1 signal. If the ectopic activation of MPK-1 is similar to a gain-of-function–like phenotype, then we should expect an enhancement of the *RAS*/*let-60* gain-of-function mutants at 20 °C by the *rskn-1*(*ok159*) allele. We tested this model and generated double mutants with *rskn-1*(*ok159*) and either *mpk-1*(*ga111*ts) or *let-60*(*ga89*ts) and assayed the oocyte numbers and morphology at 20 and 25 °C. At 20 °C, both *mpk-1*(*ga111*ts) and *let-60*(*ga89*ts) exhibit a wild-type oocyte phenotype (8, 9, 11). Although *rskn-1*(*ok159*) did not exhibit a genetic interaction with the *let-60*(*ga89ts*) gain-of-function mutation at 20 °C, we found that loss of *rskn-1* enhanced the large oocyte phenotype of *mpk-1*(*ga111*ts) at 20 °C (compare Fig. 4 *H* and *I*). Thus paradoxically, *rskn-1* must have a function to promote MPK-1 activity for its role in oocyte growth despite the observation that *rskn-1*(*ok159*) mutants display ectopic MPK-1 activation in the loop region.

At 25 °C, we observed that loss of *rskn-1* suppressed the small oocyte phenotype of *let-60*(*ga89*ts) (compare Fig. 4 *N* and *O*) but did not impact the large oocyte phenotype of *mpk-1*(*ga111*ts) (Fig. 4 *H* and *J*). These data, while surprising, suggest that an ectopic increase in dpMPK-1 upon loss of function in genes outside of the core LET-60–MPK-1 pathway is not necessarily equivalent to increased dpMPK-1 in RAS/*let-60*(*ga89*ts). These results thus present a cautionary note against making predictions on the nature of MPK-1 activation simply based on dpMPK-1 signal and suggest that orthogonal assays such as genetic or biochemical analyses, together with assessment of the dpMPK-1 pattern, will enable better determination of whether the gene functions through regulating MPK-1 directly or indirectly to lead to the phenotype.

### Ectopic MPK-1 Activation Does Not Necessarily Inhibit SC Disassembly in Diplotene Oocytes.

**Fig. 5.**
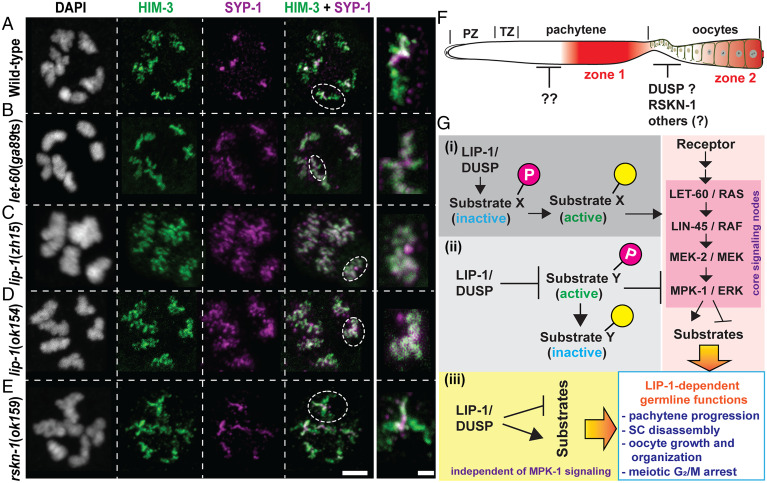
Ectopic MPK-1 activation does not necessarily inhibit SC disassembly in diplotene oocytes. (*A*–*E*) Representative confocal images of diplotene/early diakinesis germ cell from indicated genotypes labeled with anti–HIM-3 (axial element of SC, green), anti–SYP-1 (central region of SC, magenta), and DAPI (DNA, white). The dotted circles on the *Left* are shown in magnified view in the *Right*. Experiments were repeated twice; *n* = 10 gonad arms per genotype were examined, showing identical results (scale bars, 5 μm for the *Left* and 2 μm for the *Right* panels). (*F*) Schematic surface view of a hermaphroditic *C. elegans* germline displaying the activation of MPK-1 (red) and the DUSP that might control MPK-1 activity spatiotemporally. (*G*) Schematic model for LIP-1–mediated regulation of diverse biological functions in the *C. elegans* germline. Given the role of LIP-1 as an activator of MPK-1 pathway, it is plausible that LIP-1 (*i*) dephosphorylates and activate its substrate(s), which in turn triggers activation of MPK-1 pathway; (*ii*) dephosphorylates and inactivates its substrate(s) to block the activation of MPK-1 pathway; and (*iii*) might regulate a diverse group of unknown substrates, independent of the MPK-1 pathway, which execute LIP-1–mediated germline functions.

In wild-type *C. elegans* diplotene oocytes, SC central proteins are removed from the long arm of chromosomes to enable accurate chromosome segregation (21). Spatially and temporally concordant with the removal of SC central proteins from the long arm of chromosomes, dpMPK-1 is inactivated in the loop region. Nadarajan et al. (10) observed that in RAS/*let-60*(*ga89*ts) gain-of-function mutant germlines, SC central proteins are retained on the long arm, suggesting that ectopic MPK-1 activation in the loop region of the germline is sufficient to cause retention of the SC central proteins. The authors also observed that loss of *lip-1* leads to retention of the SC central protein to the long chromosomal arm (10). Because we observed that loss of *lip-1* did not lead to ectopic activation of MPK-1, we assayed for retention of the SC central protein in *lip-1* mutants, *let-60*(*ga89*ts) gain-of-function mutant, and *rskn-1*(*ok159*) mutant (which presents with ectopic dpMPK-1 signal), using HIM-3 (an axial element marker) and SYP-1 (a central protein) antibodies (Fig. 5 *A*–*E*). Consistent with the data from Nadarajan et al. (10), we observed that in both *let-60*(*ga89*ts) and *lip-1* mutants, SYP-1 is retained on the long arm of the chromosomes and colocalizes with HIM-3 signal (Fig. 5 *B*–*D*). However, we observed that SYP-1 was removed normally from the long arm of diplotene chromosomes in *rskn-1*(*ok159*) mutants (Fig. 5*E*), even though *rskn-1* mutants display ectopic MPK-1 activation in diplotene oocytes (Fig. 4*C*). Thus, we conclude that the disassembly of the SC central proteins in diplotene chromosomes likely occurs through multiple mechanisms, only some of which are influenced by MPK-1–dependent phosphorylation.

### How Might LIP-1 Regulate the Diverse Biological Outcomes in the *C. elegans* Germline?

Identification of a DUSP that opposes the bimodal activation of MPK-1 in the *C. elegans* germline remains an open question for now (Fig. 5*F*). While genetic evidence suggests that LIP-1 functions as an MPK-1 DUSP during vulval development (17), several lines of evidence presented here indicate that LIP-1 does not function as an MPK-1 DUSP in the germline. 1) Loss of *lip-1* does not lead to an increase in dpMPK-1 in dissected germlines. 2) Loss of *lip-1* does not rescue loss of *mpk-1*; in fact, when combined with an *mpk-1* reduction-of-function mutation, loss of *lip-1* leads to a further reduction in MPK-1 signaling and enhances the loss-of-function mutant phenotype. 3) In a feminized germline, loss of *lip-1* does not lead to an increase in the dpMPK-1 signal in oocytes, both in the presence and absence of the sperm signal.

We also uncovered further complexities in the regulation of the LET-60–MPK-1 pathway, which question drawing a straight line between an increase in the dpMPK-1 signal and generation of a mutant phenotype, such as the formation of small abnormal oocytes. 1) We discovered that ectopic dpMPK-1 signal in the loop region, as observed in *rskn-1*(*ok159*) mutants, is not sufficient to lead to an increase in oocyte numbers. 2) Ectopic dpMPK-1 signal in the loop region does not necessarily indicate that an allele is a gain-of-function in the MPK-1 pathway, since *rskn-1*(*ok159*) suppressed rather than enhanced the *let-60*(*ga89*ts) gain-of-function allele, even though each of the single alleles generate ectopic dpMPK-1 signal in the loop region. 3) MPK-1–dependent and independent mechanisms influence the disassembly of the SC central region on chromosomal long arms. 4) We observed that phenotypic similarities can be misleading, such as those between *ooc-5* mutant alleles and *let-60*(*ga89*ts)—both produce increased numbers of small abnormal oocytes, but *ooc-5* mutant germlines do not display ectopic or increased dpMPK-1 signal. Altogether, our results emphasize caution against overinterpretation of the mechanistic underpinnings of orthologous phenotypes produced by mutations in diverse genes within an organism or even occurring in different cell types within that species. Likewise, this caveat also applies to the interpretation of phenotypes emanating from mutations in genes conserved between different species, since each of these phenotypes may result from independent mechanisms. Finally, our analysis also provides a framework for characterizing the as yet unknown and likely distinct molecular targets through which LIP-1 may mediate its several germline functions.

During this reinvestigation, we observed that while LIP-1 does not function as an MPK-1 DUSP, nevertheless *lip-1* has an essential function in germline development and regulates several biological processes. It is likely that LIP-1 functions through yet unknown substrates (likely more than one) that are regulated to mediate its diverse functions. Here, we propose three possible models of how LIP-1 might function in the germline (Fig. 5*G*). It is likely that LIP-1 activates the MPK-1 pathway, probably indirectly, based on our observation that loss of *lip-1* results in decreased dpMPK-1 signal. Thus, functioning as a phosphatase, LIP-1 might dephosphorylate and activate its substrate(s), which in turn may positively regulate the MPK-1 pathway. Alternatively, LIP-1 may dephosphorylate and inactivate its substrate(s), which negatively regulate the MPK-1 pathway (Fig. 5 *G i* and *ii*). Critically, some of the cellular functions of LIP-1 might be regulated by substrates completely independent of the MPK-1 pathway (Fig. 5 *G iii*). Given the number of diverse biological processes regulated by LIP-1, it is quite possible that all the aforementioned possibilities are involved. To understand the role of this complex phosphosignaling network in germline physiology, further work is warranted.

## Materials and Methods

### *C. elegans* Strains, Genetics, and Maintenance.

The *C. elegans* N2 Bristol strain was used as the wild-type background, and all strains (*SI Appendix*, Table S1) were maintained at 20 °C on plates made from standard nematode growth medium as described in (31). OP50 *Escherichia coli* was used as the food source. All experiments were performed on well-fed animals. The temperature-shift experiments were performed by placing mid-L4 animals from indicated genotypes at 25 °C for 24 h. A parallel set of control experiments with the genotypes was conducted at 20 °C each time.

### Germline Dissection, Immunofluorescence Staining, and Microscopy.

After each experiment, worms (*n* = 50 to 60) were dissected and stained as described (32). The antibodies used here are shown in *SI Appendix*, Table S2. The staining was performed as described previously (11). In brief, dissected worms were fixed in 3% formaldehyde with 100 mM K_2_HPO_4_ (pH 7.2) for 10 min at room temperature, washed with phosphate-buffered saline and 0.1% Tween 20 (PBST), and postfixed with 100% methanol (−20 °C) for 1 h (for dpMPK-1 and associated rabbit antibodies, see the next paragraph) or overnight (for HIM-3 and SYP-1 coimmunostaining). Fixed gonads, in batches, were washed with PBST and blocked with 30% normal goat serum for 1 h at room temperature (for dpMPK-1 antibodies) or 3 h [for p-HTP-1(S325) antibody] before incubation with the desired primary antibody. For SYP-1 (1:1,000), p-HTP-1(S325) (1:400)–treated germ lines were incubated in primary antibody overnight at 4 °C and processed.

For staining the germline with anti–dpMPK-1, 50 to 60 worms were dissected within 3 to 5 min for each genotype and fixed immediately. After blocking the germlines with 30% normal goat serum for 1 h at room temperature, different genotypes were marked by different rabbit antibodies for each set of experiment [wild-types – HIM-8 (1:2,500), *lip-1*(*zh15*) and *rskn-1*(*ok159*) ‒ GLD-1 (1:200), *lip-1*(*ok154*) ‒ RME-2 (1:600), *mpk-1*(*ga111*ts) ‒ ANI-2 (1:400), *mpk-1*(*ga111*ts)*; lip-1*(*zh15*) and *fog-2*(*q71*) ‒ CGH-1 (1:500), *mpk-1*(*ga111*ts)*; lip-1*(*ok154*), *ooc-5*(*tn1757*) and *lip-1*(*zh15*)*; fog-2*(*q71*) ‒ SYP-1 (1:1,000)], followed by ×3 wash with PBST. All the genotypes used in an experiment were then pooled in the same tube for dpMPK-1 antibody (1:400) incubation for overnight at 4 °C and processed as described (32), followed by secondary antibody staining for anti-rabbit (which marks each of the individual marker antibodies) and anti-mouse for dpMPK-1. The slides were made with the pooled immunostained germlines. Wild-type germlines on each slide was used to set the acquisition time for dpMPK-1 accumulation. Images were acquired with a Zeiss Axio Imager M2 equipped with an AxioCam MRm camera (Zeiss, Thornwood, NY). All images were obtained as a montage at 40× with a numerical aperture 0.65 with overlapping cell boundaries and processed with Fiji software. The montages were then assembled in Adobe Photoshop CS3 and processed identically.

### Statistical Analysis.

Statistical analysis of data was done using GraphPad Prism 7.0 software. *P* values were derived using the nonparametric Mann-Whitney *U* test. *P* values lower than 0.05 were considered statistically significant. Data graphs were plotted in GraphPad Prism using the scatter dot plots or bar graph displaying the SD from the mean value. Each experiment was repeated at least three times, unless otherwise noted. *P* values are shown by brackets that indicate the groups being compared, and n values for each experiment are denoted in the corresponding figure panel or in the bar graph.

## Supplementary Material

Supplementary File

## Data Availability

All data generated in this study are included in the article main text or *SI Appendix*. All worm strains generated as part of the study will be deposited with Caenorhabditis Genetics Center (CGC) for distribution.

## References

[1] R. Roskoski Jr., ERK1/2 MAP kinases: Structure, function, and regulation. Pharmacol. Res. 66, 105–143 (2012). 22569528 10.1016/j.phrs.2012.04.005

[2] A. G. Turjanski, J. P. Vaqué, J. S. Gutkind, MAP kinases and the control of nuclear events. Oncogene 26, 3240–3253 (2007). 17496919 10.1038/sj.onc.1210415

[3] P. P. Roux, J. Blenis, ERK and p38 MAPK-activated protein kinases: A family of protein kinases with diverse biological functions. Microbiol. Mol. Biol. Rev. 68, 320–344 (2004). 15187187 10.1128/MMBR.68.2.320-344.2004 PMC419926

[4] J. Pouysségur, V. Volmat, P. Lenormand, Fidelity and spatio-temporal control in MAP kinase (ERKs) signalling. Biochem. Pharmacol. 64, 755–763 (2002). 12213567 10.1016/s0006-2952(02)01135-8

[5] K. Kondoh, E. Nishida, Regulation of MAP kinases by MAP kinase phosphatases. Biochim. Biophys. Acta 1773, 1227–1237 (2007). 17208316 10.1016/j.bbamcr.2006.12.002

[6] C. J. Caunt, S. M. Keyse, Dual-specificity MAP kinase phosphatases (MKPs): Shaping the outcome of MAP kinase signalling. FEBS J. 280, 489–504 (2013). 22812510 10.1111/j.1742-4658.2012.08716.x PMC3594966

[7] M. A. Miller , A sperm cytoskeletal protein that signals oocyte meiotic maturation and ovulation. Science 291, 2144–2147 (2001). 11251118 10.1126/science.1057586

[8] M.-H. Lee , Multiple functions and dynamic activation of MPK-1 extracellular signal-regulated kinase signaling in *Caenorhabditis elegans* germline development. Genetics 177, 2039–2062 (2007). 18073423 10.1534/genetics.107.081356 PMC2219468

[9] S. Arur , Multiple ERK substrates execute single biological processes in *Caenorhabditis elegans* germ-line development. Proc. Natl. Acad. Sci. U.S.A. 106, 4776–4781 (2009). 19264959 10.1073/pnas.0812285106 PMC2660749

[10] S. Nadarajan , The MAP kinase pathway coordinates crossover designation with disassembly of synaptonemal complex proteins during meiosis. eLife 5, e12039 (2016). 26920220 10.7554/eLife.12039 PMC4805554

[11] D. Das, S.-Y. Chen, S. Arur, ERK phosphorylates chromosomal axis component HORMA domain protein HTP-1 to regulate oocyte numbers. Sci. Adv. 6, eabc5580 (2020). 33127680 10.1126/sciadv.abc5580 PMC7608811

[12] B. D. Page, S. Guedes, D. Waring, J. R. Priess, The *C. elegans* E2F- and DP-related proteins are required for embryonic asymmetry and negatively regulate Ras/MAPK signaling. Mol. Cell 7, 451–460 (2001). 11463371 10.1016/s1097-2765(01)00193-9

[13] M. A. Miller, P. J. Ruest, M. Kosinski, S. K. Hanks, D. Greenstein, An Eph receptor sperm-sensing control mechanism for oocyte meiotic maturation in *Caenorhabditis elegans*. Genes Dev. 17, 187–200 (2003). 12533508 10.1101/gad.1028303 PMC195972

[14] D. L. Church, K.-L. Guan, E. J. Lambie, Three genes of the MAP kinase cascade, *mek-2, mpk-1/sur-1* and *let-60 ras*, are required for meiotic cell cycle progression in *Caenorhabditis elegans*. Development 121, 2525–2535 (1995). 7671816 10.1242/dev.121.8.2525

[15] T. L. Gumienny, E. Lambie, E. Hartwieg, H. R. Horvitz, M. O. Hengartner, Genetic control of programmed cell death in the *Caenorhabditis elegans* hermaphrodite germline. Development 126, 1011–1022 (1999). 9927601 10.1242/dev.126.5.1011

[16] R. Rutkowski , Regulation of *Caenorhabditis elegans* p53/CEP-1-dependent germ cell apoptosis by Ras/MAPK signaling. PLoS Genet. 7, e1002238 (2011). 21901106 10.1371/journal.pgen.1002238 PMC3161941

[17] T. Berset, E. F. Hoier, G. Battu, S. Canevascini, A. Hajnal, Notch inhibition of RAS signaling through MAP kinase phosphatase LIP-1 during *C. elegans* vulval development. Science 291, 1055–1058 (2001). 11161219 10.1126/science.1055642

[18] A. Hajnal, T. Berset, The *C.elegans* MAPK phosphatase LIP-1 is required for the G(2)/M meiotic arrest of developing oocytes. EMBO J. 21, 4317–4326 (2002). 12169634 10.1093/emboj/cdf430 PMC126168

[19] S. E. Basham, L. S. Rose, Mutations in *ooc-5* and *ooc-3* disrupt oocyte formation and the reestablishment of asymmetric PAR protein localization in two-cell *Caenorhabditis elegans* embryos. Dev. Biol. 215, 253–263 (1999). 10545235 10.1006/dbio.1999.9447

[20] G. Huelgas-Morales, M. Sanders, G. Mekonnen, T. Tsukamoto, D. Greenstein, Decreased mechanotransduction prevents nuclear collapse in a *Caenorhabditis elegans* laminopathy. Proc. Natl. Acad. Sci. U.S.A. 117, 31301–31308 (2020). 33229589 10.1073/pnas.2015050117 PMC7733798

[21] E. Martinez-Perez , Crossovers trigger a remodeling of meiotic chromosome axis composition that is linked to two-step loss of sister chromatid cohesion. Genes Dev. 22, 2886–2901 (2008). 18923085 10.1101/gad.1694108 PMC2569886

[22] M. R. Lackner, S. K. Kim, Genetic analysis of the *Caenorhabditis elegans* MAP kinase gene *mpk-1*. Genetics 150, 103–117 (1998). 9725833 10.1093/genetics/150.1.103 PMC1460334

[23] A. Jaramillo-Lambert, M. Ellefson, A. M. Villeneuve, J. Engebrecht, Differential timing of S phases, X chromosome replication, and meiotic prophase in the *C. elegans* germ line. Dev. Biol. 308, 206–221 (2007). 17599823 10.1016/j.ydbio.2007.05.019

[24] Z. Qin, E. J. A. Hubbard, Non-autonomous DAF-16/FOXO activity antagonizes age-related loss of *C. elegans* germline stem/progenitor cells. Nat. Commun. 6, 7107 (2015). 25960195 10.1038/ncomms8107 PMC4432587

[25] P. E. Kuwabara, M.-H. Lee, T. Schedl, G. S. Jefferis, A *C. elegans* patched gene, *ptc-1*, functions in germ-line cytokinesis. Genes Dev. 14, 1933–1944 (2000). 10921907 PMC316821

[26] J. McCarter, B. Bartlett, T. Dang, T. Schedl, Soma-germ cell interactions in *Caenorhabditis elegans*: Multiple events of hermaphrodite germline development require the somatic sheath and spermathecal lineages. Dev. Biol. 181, 121–143 (1997). 9013925 10.1006/dbio.1996.8429

[27] R. Clifford , FOG-2, a novel F-box containing protein, associates with the GLD-1 RNA binding protein and directs male sex determination in the *C. elegans* hermaphrodite germline. Development 127, 5265–5276 (2000). 11076749 10.1242/dev.127.24.5265

[28] A. L. Lopez III , DAF-2 and ERK couple nutrient availability to meiotic progression during *Caenorhabditis elegans* oogenesis. Dev. Cell 27, 227–240 (2013). 24120884 10.1016/j.devcel.2013.09.008 PMC3829605

[29] S. Nadarajan, J. A. Govindan, M. McGovern, E. J. A. Hubbard, D. Greenstein, MSP and GLP-1/Notch signaling coordinately regulate actomyosin-dependent cytoplasmic streaming and oocyte growth in *C. elegans*. Development 136, 2223–2234 (2009). 19502484 10.1242/dev.034603 PMC2729341

[30] R. A. Green , A high-resolution *C. elegans* essential gene network based on phenotypic profiling of a complex tissue. Cell 145, 470–482 (2011). 21529718 10.1016/j.cell.2011.03.037 PMC3086541

[31] S. Brenner, The genetics of *Caenorhabditis elegans*. Genetics 77, 71–94 (1974). 4366476 10.1093/genetics/77.1.71 PMC1213120

[32] A. L. Gervaise, S. Arur, Spatial and temporal analysis of active ERK in the *C. elegans* germline. J. Vis. Exp. 117, 54901 (2016). 10.3791/54901 PMC5226324 27929466

