# Supplementary material for: Reevaluation of the role of LIP-1 as an ERK/MPK-1 dual specificity phosphatase in the C. elegans germline
Source: Proc Natl Acad Sci U S A. 2022 Jan 12;119(3):e2113649119. doi: 10.1073/pnas.2113649119 (PMC8784128; doi:10.1073/pnas.2113649119)
Supplement: Supplementary File [file pnas.2113649119.sapp.pdf]

Supplementary Information

Reevaluation of the role of LIP-1 as ERK/MPK-1 dual specificity phosphatase in the *C. elegans* germline

Debabrata Das[1], Jacob Seemann[1], David Greenstein[2], Tim Schedl[3], Swathi Arur[1]*

[1] Department of Genetics, UT MD Anderson Cancer Center, Houston, TX 77030, USA.

[2] Department of Genetics, Cell Biology, and Development, University of Minnesota, Minneapolis, MN 55455

[3] Department of Genetics, Washington University School of Medicine, St. Louis, MO 63110

*Corresponding author: Swathi Arur
Email: sarur@mdanderson.org

This PDF file includes:

Supplementary text
Figures S1 to S7
Table S1 to S2
SI References

Supplementary Information Text

Materials and Methods

Live imaging of worms

Five to seven animals, each time, were mounted on 2% agarose pads with 10 µl of 0.1 M levamisole in M9 and imaged with Zeiss Axio Imager M2 equipped with an AxioCam MRm camera (Zeiss). All images were obtained using AxioVision software (Zeiss) as a montage of images at 40x with numerical aperture of 0.65.

Progeny assay and embryonic lethality

Ten mid-L4-stage hermaphroditic worms of the indicated genotypes were placed individually on plates. The plates were placed either at 20ºC or shifted to 25ºC, according to the experiment. The parent worm was moved to a fresh plate after every 12 to 18 hours, and the total number of embryos was counted on the original plate. This process was repeated over 5 to 7 days until the worms stopped producing progeny. The total number of embryos (across the entire lay period) was added up for each animal. Similarly, adult progeny was counted from each of those plates after 2-3 days and added for each animal. Embryonic lethality was calculated in percentage as follows [(number of embryos − number of adults)/numbers of embryos] × 100. The analysis was performed in three to five replicates.

Pachytene progression, oocyte number and Emo phenotype

Dissection and DAPI staining were used for analysis of pachytene progression, oocyte number and Emo phenotype. For each of the experiments, 50-60 mid-L4 worms of the indicated genotypes were placed in a single plate and kept either at 20ºC or shifted to 25ºC for 24 hours. The worms were then dissected and fixed in 3% formaldehyde with 100 mM K_2HPO_4 (pH 7.2) for 10 min at room temperature washed with phosphate-buffered saline (PBS) and 0.1% Tween 20 (PBST) and post fixed with 100% methanol (−20°C) for 1 hour. The germlines were stained with DAPI (1:1000, stock: 1 µg/mL) for 30 min at room-temperature and processed for slide preparation. The images were captured with Zeiss (Thornwood, NY) Axio Imager M2 equipped with an AxioCam MRm camera (Zeiss) with Z-stacks (0.6 µm) at 40x with numerical aperture of 0.65. Pachytene germ cells with their distinct morphology were then checked manually in each germline in the proximal gonad (after the loop region). Pachytene-progression defects were scored as the percentage of

germlines with any pachytene-stage germ cells located proximal to the loop region. Oocytes were scored using the DAPI-stained chromosomal morphology of diplotene/diakinesis stage germ cells and counted manually. The Emo phenotype is detected as large DAPI blobs (distinct from 6 bivalents at the diakinesis). Germlines were counted as either with or without Emo phenotype and expressed as percentage.

Mating assay of feminized *lip-1* mutant

Forty mid-L4-stage feminized worms of genotypes *fog-1*(*q71*) and *lip-1*(*zh15*)*; fog-1*(*q71*) were grown on culture plates for 20h at 20ºC. Approximately 60 mid-L4+24h wild-type males were introduced in each plate. The mating assay was continued for 4h at 20ºC, after which the females were removed, dissected, and stained for dpMPK-1 analysis. Staining and dissection was performed as described in main text. Because dpMPK-1 staining was performed in the same tube for both the genotypes, each genotype was first labeled with different markers. *fog-2*(*q71*) was labeled with p-SUN-1 (1:800) and *lip-1*(*zh15*)*;fog-2*(*q71*) was labeled with HTP-3 (1:800)]. Wild-type hermaphrodite (mid-L4+24h) germlines were used as a positive control. All the genotypes used in an experiment were then pooled in the same tube for rabbit dpMPK-1 antibody (1:400) and MSP antibody (1:200) incubation for overnight at 4ºC and processed as described (1), followed by secondary antibody staining for anti-Guinea pig (which marks each of the individual genotype), anti-Rabbit for dpMPK-1 and anti-Mouse for detecting MSP, to detect MSP as a marker of successful mating. The slides were made with the pooled immunostained germlines. Wild-type hermaphrodite germlines on each slide were used to set the acquisition time for dpMPK-1 accumulation. Images were acquired with Zeiss Axio Imager M2 equipped with an AxioCam MRm camera (Zeiss). All images were obtained as a montage at 40x with a numerical aperture of 0.65, with overlapping cell boundaries and processed with ImageJ software. The montages were then assembled in Adobe Photoshop CS3 and processed identically.

SYP-1 disassembly assay

For the SYP-1 disassembly assay, 50-60 mid-L4 worms/plate from each genotype were shifted to 25ºC for 24h. The animals were then dissected, and germlines were immunostained with anti-HIM-3 and anti-SYP-1. Because both the antibodies were raised against Rabbit, anti-HIM-3 was first conjugated with Alexa Fluor 488 according to the manufacturer's protocol (APEX™ antibody labeling kit, Thermo Fisher Scientific, A10468).

Dissection, fixation, post-fixation, primary SYP-1 antibody (1:1000) and secondary antibody step with anti-rabbit (1:800) were done as described (1). After completion of washing the anti-Rabbit secondary with PBST, Apex-488 conjugated HIM-3 antibody (1:20) were used for overnight incubation at 4ºC and processed. Images were acquired with an inverted laser scanning confocal microscope (Zeiss LSM 800) at ×63 with the Z-stacks (0.19 μm) and deconvolved using the AutoQuant X3 deconvolution software. Images were processed in Fiji (ImageJ). The stacks of DAPI, HIM-3, and SYP-1 were projected maximally and overlaid in Adobe Photoshop to display the figures presented.

Figure S1

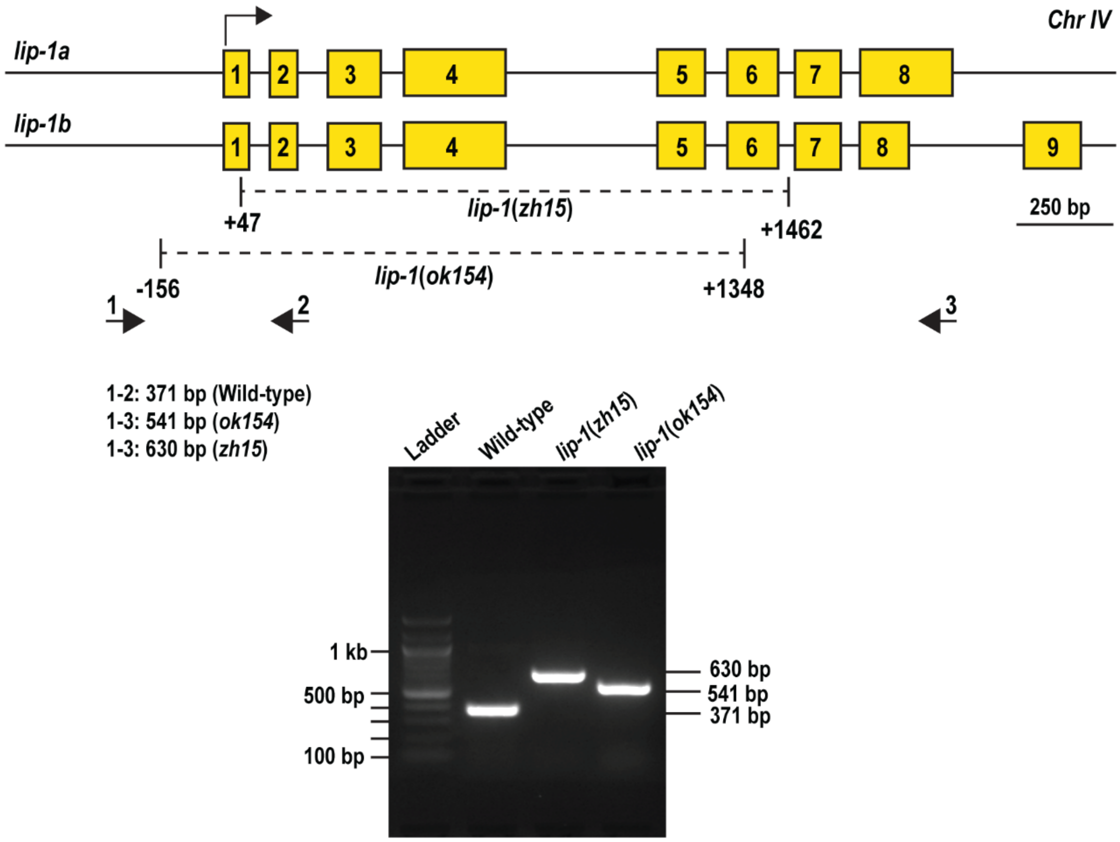

Figure S1. Gene structure and description of *lip-1* alleles used in the study. Intron-exon structure of the two isoforms of *lip-1*. Each numbered yellow box represents an exon. The dotted line underneath marks the *zh15* and *ok154* deletion. The numbers indicate the positions of the breakpoints (in bp) relative to the start codon (angled arrow on top of exon 1). The numbered arrows indicate the three primers used in each reaction for the PCR analysis shown in the lower panel and the size of the products.

Figure S2

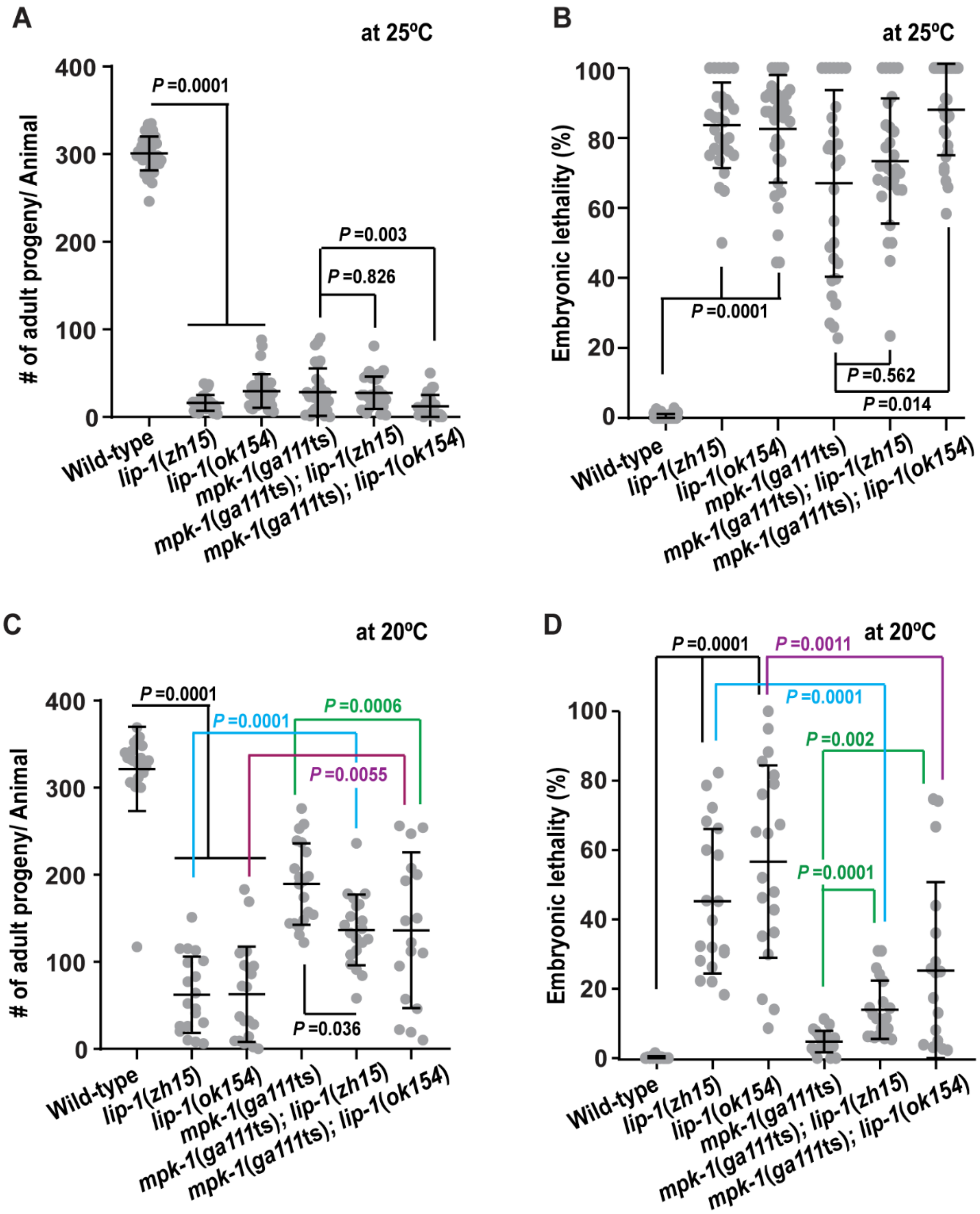

Figure S2. Loss of *lip-1* enhances *mpk-1*(*ga111*ts) sterility and embryonic lethality. Quantitative analysis for fertility (production of adult progeny) and embryonic lethality of the indicated genotypes at the indicated temperatures. Experiments were repeated three times; n = 17 to 23 per genotype at 20ºC and 29 to 42 per genotype at 25ºC. Statistical significance was

calculated using a nonparametric Mann-Whitney test, and P values are indicated between the groups compared. Error bars represent means ± SD.

Figure S3

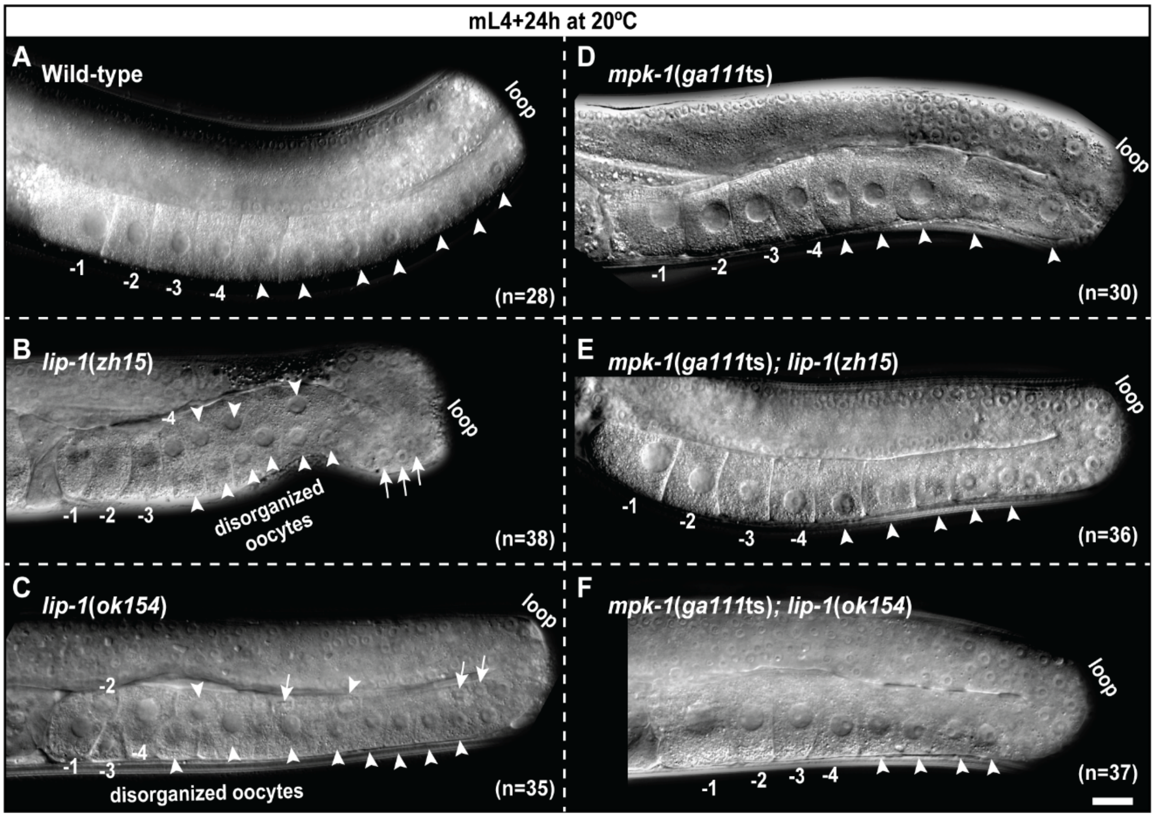

Figure S3. *mpk-1(ga111*ts*)* suppresses the *lip-1* disorganized oocyte phenotype at 20°C. Differential interference contrast (DIC) microscopy images of germlines from indicated genotypes. Germline morphology of the double mutants between *mpk-1*(*ga111*ts) and *lip-1*(*zh15*) or *lip-1*(*ok154*) are indistinguishable from the *mpk-1*(*ga111*ts) single mutant germlines at 20ºC. The loop region is on the right in the photographs and oocytes on the ventral side. Oocytes are numbered from proximal to distal polarity (toward loop). The most proximal oocyte is labeled as −1. Arrowheads indicate oocytes, arrows indicate pachytene germ cells. Experiments were repeated three times; the total number of germlines analyzed for each experiment (n) is indicated in each panel. Scale bar, 25 µm.

Figure S4

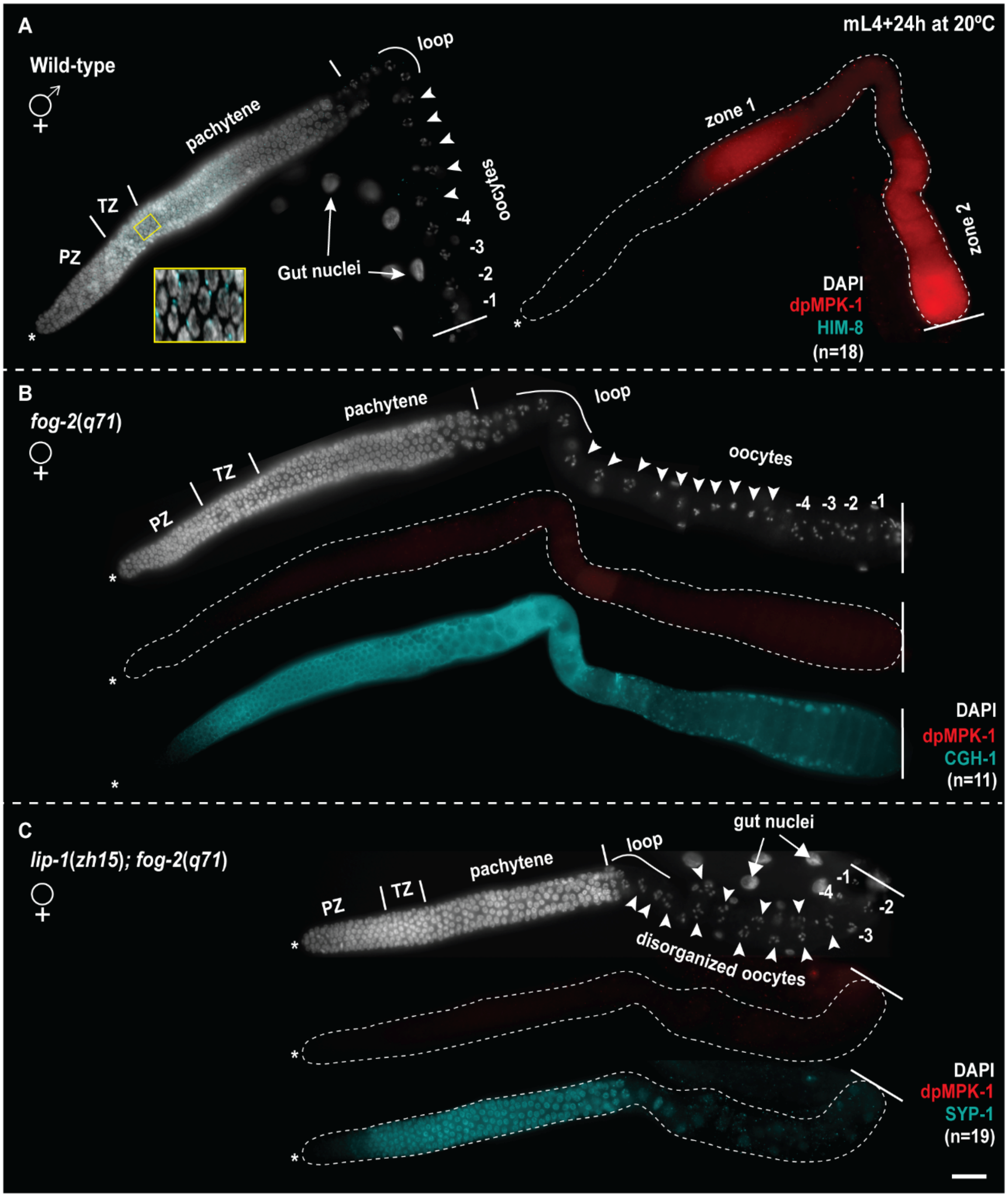

Figure S4. Loss of *lip-1* has no impact on MPK-1 activation in adult feminized germlines. Dissected germlines are shown in a distal (left, asterisks) to proximal (right) orientation. (**A-C**) Representative images of dissected germlines probed with anti-dpMPK-1 (red), genotype marking antibodies (cyan), and DAPI (DNA, white) of indicated genotypes. HIM-8 panel is overlapped with DAPI and an enlarged view of the TZ region is shown in the inset. Oocytes are numbered from

proximal to distal polarity (toward loop). The most proximal oocyte is labeled as −1. Arrowheads indicate oocytes. Experiments were repeated three times; the total number of germlines (n) /genotype examined is indicated in each panel; all showing similar dpMPK-1 staining pattern. Scale bar, 25 μm.

Figure S5

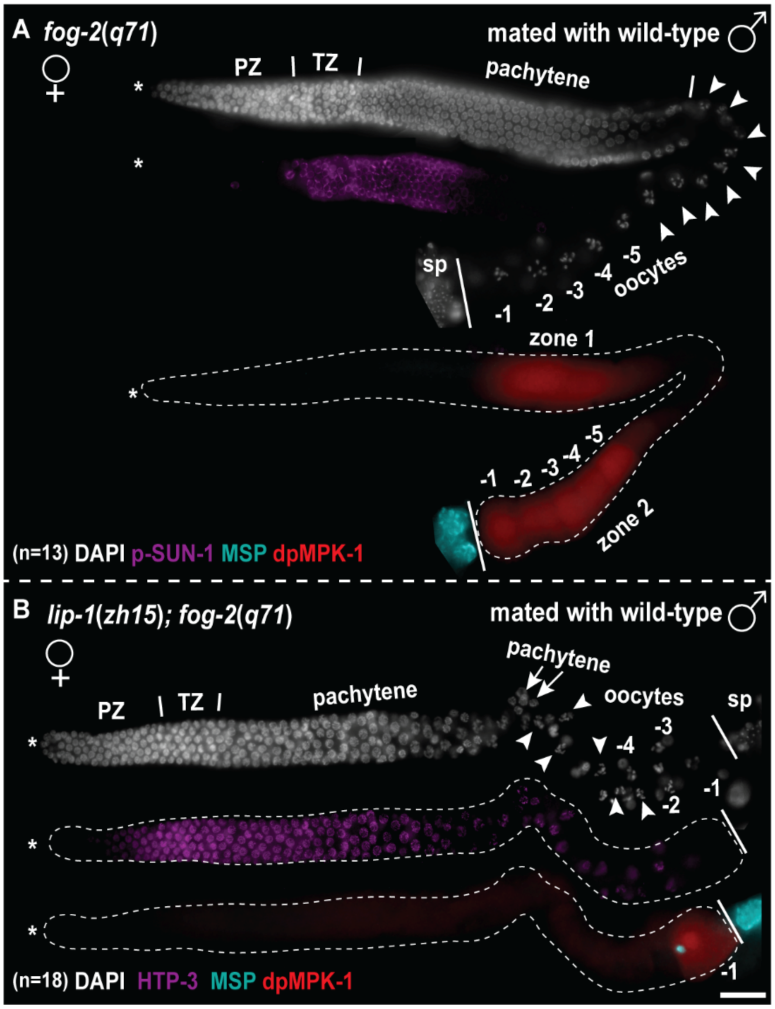

Figure S5. Mating of feminized *lip-1* animals with wild-type males does not lead to MPK-1 over-activation in the germline. The dissected germlines are displayed in a distal (left, asterisks) to proximal (right) orientation. (**A-B**) Representative images of dissected germlines probed with anti-dpMPK-1 (red), genotype marking antibodies (purple), MSP (cyan) and DAPI (DNA, white) of indicated genotypes. Oocytes are numbered from proximal to distal polarity (toward loop). The most proximal oocyte is labeled as −1. Arrowheads indicate oocytes, arrows indicate pachytene germ cells. Experiments were repeated twice; total number of germlines (n) /genotype examined is indicated in each panel; all showing similar dpMPK-1 staining pattern. sp, sperm nuclei. Scale bar, 25 μm.

Figure S6

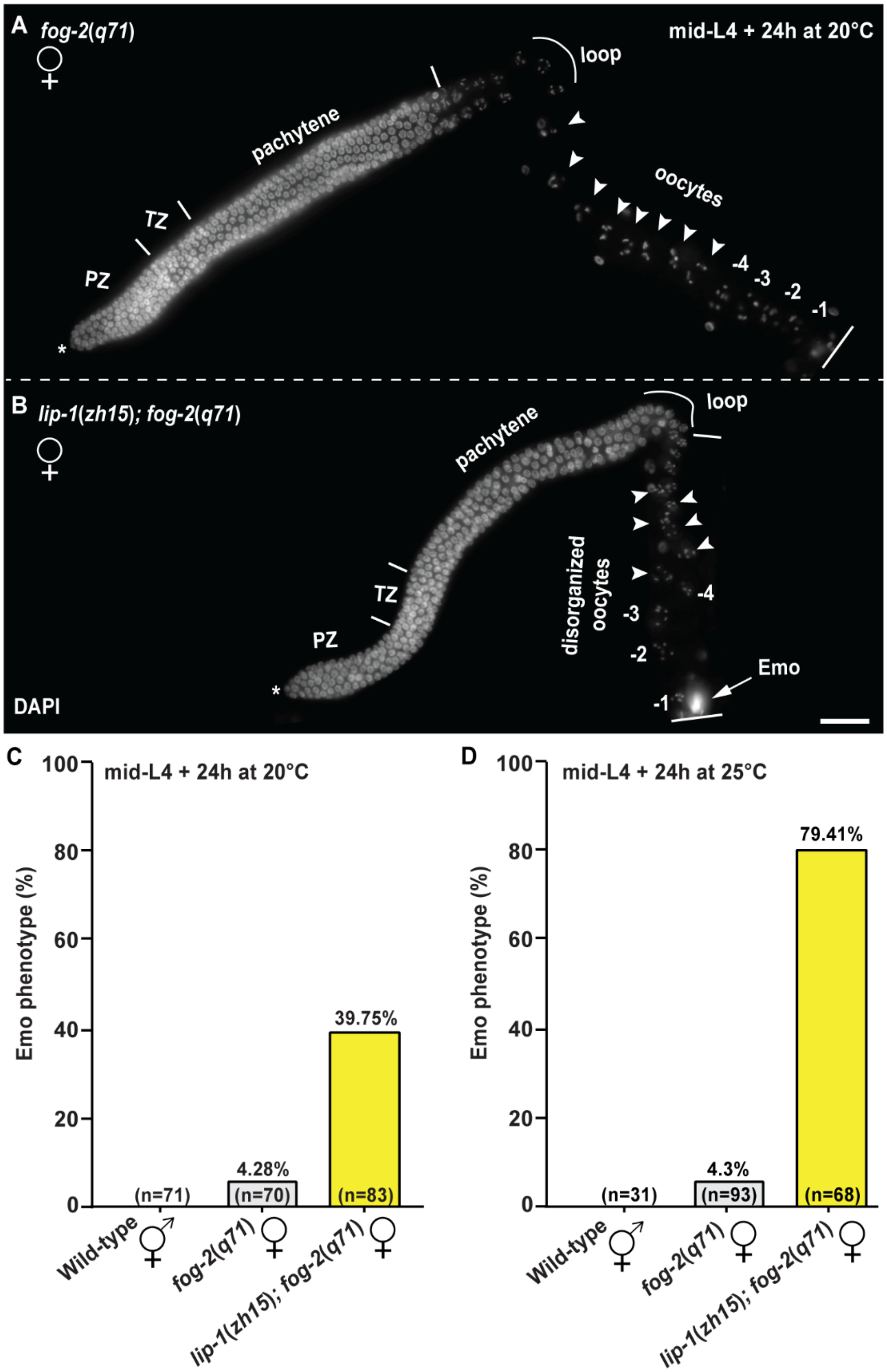

Figure S6. Feminized *lip-1* mutants are defective in holding the meiotic arrest. (**A-B**) Dissected DAPI-stained germlines of indicated genotypes displaying germline morphology. The

dissected germlines are displayed in a distal (left, asterisks) to proximal (right) orientation. Arrowheads indicate oocytes; arrow indicates an “Emo” oocyte. Scale bar, 25 μm. (**C-D**) Quantification of germlines of the indicated genotypes with the Emo phenotype expressed as a percentage (noted above the bars). Experiments were repeated thrice; the total number of germlines counted (n) is indicated on the bars.

Figure S7

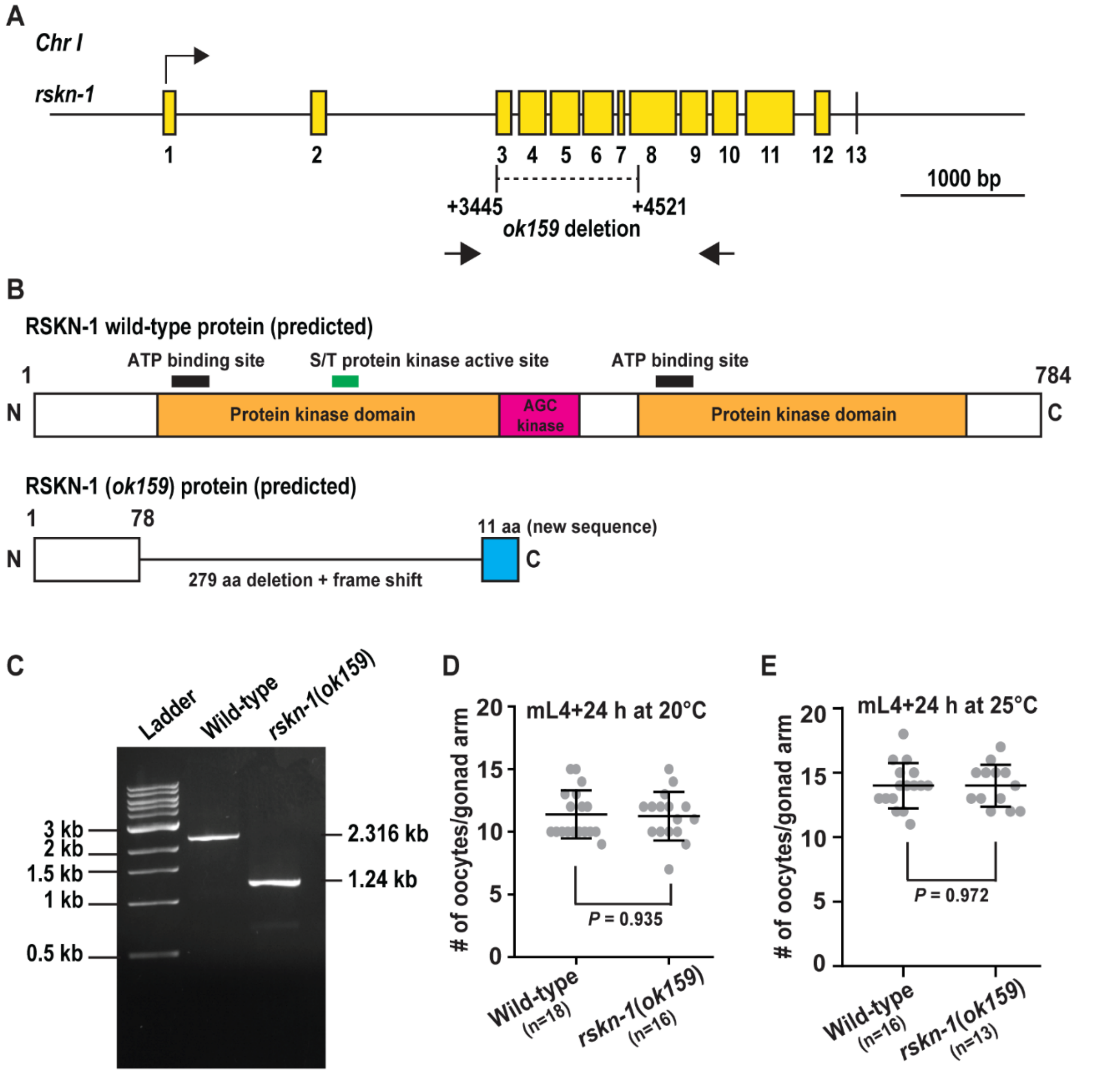

Figure S7. Description of the *rskn-1(ok159)* allele used in this study. (**A**) Intron-exon structure of the *rskn-1* gene. Each numbered yellow box represents an exon. The dotted line underneath shows the extent of the *ok159* deletion. The numbers indicate the positions of the breakpoints (in bp) relative to the start codon (angled arrow on top of exon 1). The arrows below indicate the two primers used for the PCR analysis shown in the panel C. (**B**) Predicted protein structure of wild-type RSKN-1 [1 to 784 amino acids (aa), marked at the N- and C- termini of the protein, respectively] and the predicted truncated 89 aa peptide for the *ok159* allele*.* (**C**) Agarose gel showing the PCR products generated from the wild type and the *rskn-1*(*ok159*) deletion, which removes 1076. (**D-E**) Scatter dot plot of oocyte numbers from the indicated genotypes and temperatures. The experiment was repeated twice, the total number of germlines (n) examined is indicated with the genotype. Statistical significance was calculated by a nonparametric Mann-Whitney test, and data represented as means ± SD. *P* values are noted between groups compared.

Table S1. ***C. elegans* strains.** The following *C. elegans* strains were used.

Strain name	Genotype
N2	Wild-type
BS3760	*rskn-1*(*ok159*) *I*
AUM1528	*rskn-1*(*ok159*) *I; ltIs38* [(*pAA1*) *pie-1p::GFP::PH*(*PLC1delta1*) + *unc-119*(+)] *III; ltIs37* [*pie-1p::mCherry::his-58* (*pAA64*) + *unc-119*(+)] *IV*
AUM1506b	*rskn-1*(*ok159*) *I; mpk-1*(*ga111*ts) *ltIs38* [(*pAA1*) *pie-1p::GFP::PH* (*PLC1delta1*) + *unc-119*(+)] *III; ltIs37* [*pie-1p::mCherry::his-58*(*pAA64*) + *unc-119*(+)] *IV*
AUM1504b	*rskn-1*(*ok159*) *I; ltIs38* [(*pAA1*) *pie-1p::GFP::PH*(*PLC1delta1*) + *unc-119*(+)] *III; let-60*(*ga89*ts) *ltIs37*[*pie-1p::mCherry::his-58*(*pAA64*) + *unc-119*(+)] *IV*
DG4309	*ooc-5*(*tn1757*)/ *mIn1*[*dpy-10*(*e128*)*mIs14*] *II*
BS3364	*mpk-1*(*ga111*ts) *III*
AUM1034	*ltIs38* [(*pAA1*) *pie-1p::GFP::PH*(*PLC1delta1*) + *unc-119*(+)] *III; ltIs37* [*pie-1p::mCherry::his-58* (*pAA64*) + *unc-119*(+)] *IV*
AUM1038	*mpk-1*(*ga111*ts) *ltIs38* [(*pAA1*) *pie-1p::GFP::PH*(*PLC1delta1*) + *unc-119*(+)] *III; ltIs37* [*pie-1p::mCherry::his-58* (*pAA64*) + *unc-119*(+)] *IV*
AUM1722	*mpk-1*(*ga111*ts) *III; lip-1*(*zh15*) *IV*
BS3745	*mpk-1*(*ga111*ts) *III; lip-1*(*ok154*) *IV*
AUM1230	*ltIs38* [(*pAA1*) *pie-1p::GFP::PH*(*PLC1delta1*) + *unc-119*(+)] *III; let-60*(*ga89*ts) *ltIs37* [*pie-1p::mCherry::his-58*(*pAA64*) + *unc-119*(+)] *IV*
AH102	*lip-1*(*zh15*) *IV*
BS3727	*lip-1*(*ok154*) *IV*
DG1737	*lip-1*(*zh15*) *IV; fog-2*(*q71*) *V*
AUM1606	*htp-1*(*viz62* [*S325A*]) *IV*
CB4108	*fog-2*(*q71*) *V*

Table S2. Antibodies. The following antibodies were used in this study.

Antibodies	Source, Identifier (reference)
dpMPK-1 [anti-MAPKYT]	Sigma-Aldrich, MO, M8159 (1-4)
dpMPK-1 [Phospho-p44/42 MAPK (Erk1/2)]	Cell Signaling, MA, #9101
p-HTP-1(S325)	Arur Lab (2)
RME-2	B. Grant (5)
GLD-1	Schedl Lab (6)
SYP-1	N. Silva (7)
HIM-3	Novus, MO, 53470002 (2)
CGH-1	Greenstein Lab (3)
HIM-8	Novus, MO, 41980002
ANI-2	K. Oegema (8)
p-SUN-1(S8)	V. Jantsch (2)
HTP-3	Y. Kim (9)
MSP	Greenstein Lab
Goat–anti-mouse Alexa Fluor 555	Invitrogen, CA, A-21422
Goat–anti-rabbit Alexa Fluor 488	Invitrogen, CA, A-11008
Goat–anti-guinea pig Cy5	Abcam, MA, ab102372

SI References

1. A. L. Gervaise, S. Arur, Spatial and temporal analysis of active ERK in the *C. elegans* germline. *Journal of visualized experiments: JoVE* **117**, 54901 (2016).
2. D. Das, S.-Y. Chen, S. Arur, ERK phosphorylates chromosomal axis component HORMA domain protein HTP-1 to regulate oocyte numbers. *Science advances* **6**, eabc5580 (2020).
3. M.-H. Lee *et al.*, Multiple functions and dynamic activation of MPK-1 extracellular signal-regulated kinase signaling in *Caenorhabditis elegans* germline development. *Genetics* **177**, 2039-2062 (2007).
4. S. Arur *et al.*, Multiple ERK substrates execute single biological processes in *Caenorhabditis elegans* germ-line development. *Proceedings of the National Academy of Sciences* **106**, 4776-4781 (2009).
5. B. Grant, D. Hirsh, Receptor-mediated endocytosis in the *Caenorhabditis elegans* oocyte. *Molecular Biology of the Cell* **10**, 4311-4326 (1999).
6. A. R. Jones, R. Francis, T. Schedl, GLD-1, a cytoplasmic protein essential for oocyte differentiation, shows stage-and sex-specific dxpression during *Caenorhabditis elegans* germline development. *Developmental Biology* **180**, 165-183 (1996).
7. E. Janisiw *et al.*, Poly (ADP-ribose) glycohydrolase coordinates meiotic DNA double-strand break induction and repair independent of its catalytic activity. *Nature Communications* **11**, 1-15 (2020).
8. A. S. Maddox, B. Habermann, A. Desai, K. Oegema, Distinct roles for two *C. elegans* anillins in the gonad and early embryo. *Development* **132**, 2837–2848 (2005).
9. M. A. Hurlock *et al.*, Identification of novel synaptonemal complex components in *C. elegans. Journal of Cell Biology* **219**, e201910043 (2020).
